# CAR‐Engineered Cell Therapies Beyond Cancer: Reprogramming Fibrosis and Immune‐Mediated Inflammation

**DOI:** 10.1002/advs.77503

**Published:** 2026-08-30

**Authors:** Peng Jun Xu, Zhou Jing Zhang, Jing Jin, Hai Yuan Xu, Ya Xin Gu, Qian Qian Yang, You Lang Zhou

**Affiliations:** ^1^ The Nanomedicine Research Laboratory Hand Surgery Research Center Research Central of Clinical Medicine Affiliated Hospital of Nantong University Medical School of Nantong University Nantong China; ^2^ Department of Stomatology Affiliated Lianyungang Clinical College of Nantong University Lianyungang China; ^3^ Center for Rehabilitation Medicine Department of Rehabilitation Zhejiang Provincial People's Hospital Affiliated People's Hospital Hangzhou Medical College Hangzhou Zhejiang China

**Keywords:** autoimmune diseases, CAR‐M, CAR‐NK, CAR‐T, fibrosis, inflammation

## Abstract

Chimeric antigen receptor (CAR)‐engineered cell therapies are being extended from hematological malignancies to autoimmune, inflammatory, and fibrotic diseases, although the maturity of evidence differs markedly among platforms and indications. Early clinical evidence from case reports, small case series, and early‐phase trials suggests that CD19‐ or BCMA‐directed CAR‐T cells can induce deep B‐cell or plasma‐cell depletion and sustained remission in selected patients with refractory autoimmune diseases; however, their comparative efficacy, durability, and long‐term safety remain incompletely defined. By contrast, CAR‐based treatment of organ fibrosis remains predominantly preclinical. In animal models, transient in vivo CAR‐T generation has attenuated fibrosis, whereas CAR‐macrophage(CAR‐M) approaches have demonstrated targeted phagocytosis and microenvironmental remodeling. Evidence for CAR‐natural killer cells (CAR‐NK) in non‐oncological diseases is currently limited to preclinical studies and an individual‐patient observation, despite their potential advantages for allogeneic and off‐the‐shelf manufacturing. This Review critically compares CAR‐T, CAR‐M, and CAR‐NK platforms, examines emerging unconventional immune‐cell and iPSC‐derived products, and evaluates programmable and in vivo CAR‐engineering strategies. We propose “controllable spatiotemporal reprogramming” as a framework linking target specificity, tissue distribution, activity duration, reversibility, manufacturing, and disease‐specific safety requirements.

## Graphical abstract

CAR‐engineered cell therapies are expanding beyond cancer toward immune resetting, pathological‐cell clearance, matrix remodeling, and microenvironmental reprogramming in autoimmune, inflammatory, and fibrotic diseases. This Review compares CAR‐T, CAR‐macrophage, and CAR‐NK platforms and proposes controllable spatiotemporal reprogramming to align target selection, tissue access, activity duration, reversibility, manufacturing, and safety with disease‐specific therapeutic needs.

## 1 Introduction

Chimeric antigen receptor (CAR)‐engineered cell therapy has reshaped the treatment landscape of hematological malignancies by enabling major histocompatibility complex‐independent antigen recognition and potent effector‐cell activation. Since the approval of the first CD19‐targeted CAR‐T product in 2017, CAR‐T therapies have achieved remarkable clinical responses in B‐cell malignancies and multiple myeloma. However, the therapeutic logic of CAR technology is now expanding beyond direct tumor‐cell killing [1]. Current evidence in refractory autoimmune diseases, particularly CD19‐directed CAR‐T therapy for systemic lupus erythematosus, derives mainly from case reports, small case series, and early‐phase clinical studies [2, 3]. These preliminary clinical findings suggest that CAR‐engineered cells may induce deep depletion of pathogenic immune‐cell populations and promote immune resetting, but their comparative efficacy, durability, and long‐term safety have not yet been established in large controlled trials.

Fibrosis and inflammatory autoimmune diseases represent two major disease contexts in which such a strategy may be particularly relevant. Fibrosis is a common final pathway of chronic organ injury, characterized by excessive extracellular matrix deposition and progressive loss of normal tissue architecture [4, 5], whereas autoimmune diseases are driven by persistent immune dysregulation and recurrent systemic tissue damage [6, 7]. Current anti‐fibrotic therapies mainly delay disease progression rather than reverse established fibrosis, and conventional immunosuppressive regimens often require long‐term administration with risks of relapse, infection, and malignancy. Importantly, inflammation and fibrosis are not independent processes; chronic inflammatory signaling promotes fibroblast activation and extracellular matrix deposition, while dense fibrotic matrix further sustains local immune dysfunction. This reciprocal loop provides a rationale for therapeutic strategies that can simultaneously target pathogenic immune drivers and remodel diseased tissue microenvironments [8, 9]. In contrast to the emerging clinical evidence in autoimmune diseases, CAR‐based therapy for fibrosis remains supported predominantly by in vitro studies and animal models and should therefore be regarded as preclinical proof of concept rather than evidence of established clinical efficacy.

With the extension of CAR technology from T cells to other immune‐cell lineages, CAR‐T cells, CAR‐macrophages (CAR‐M), and CAR‐natural killer cells (CAR‐NK) have emerged as functionally complementary platforms [10]. Their translational maturity, however, differs substantially. CAR‐T cells have generated early clinical evidence in refractory autoimmune diseases, mainly from case reports, small case series, and early‐phase trials, whereas their application to fibrosis remains preclinical. CAR‐M therapies for fibrotic and inflammatory diseases are currently supported primarily by in vitro and animal studies. Non‐oncological CAR‐NK development also remains predominantly preclinical, with only limited individual‐patient observations reported to date. Accordingly, throughout this Review, we distinguish among regulatory approval, prospective clinical investigation, case reports or small case series, individual‐patient observations, and preclinical evidence when assessing therapeutic progress. Therefore, the goal of CAR therapy in non‐oncological diseases is not simply to enhance cytotoxicity, but to achieve controllable reprogramming of pathological immune–fibrotic processes [11]. Building on previous reviews that have largely focused on individual CAR platforms or specific disease categories, this Review provides an integrated comparison of CAR‐T, CAR‐M, and CAR‐NK cells across fibrotic and immune‐mediated inflammatory diseases. Its distinctive contribution is to combine this three‐platform analysis with a controllable spatiotemporal reprogramming framework, while placing particular emphasis on fibrosis‐specific targets, tissue microenvironments, and translational barriers.

## 2 Design Principles and Target‐Selection Logic of CAR‐Engineered Cell Therapy

### 2.1 Evolution of CAR Structures Across Five Generations

The modular architecture of CARs provides the structural basis for their application across different effector‐cell types and disease contexts [12]. A canonical CAR contains four core components: an extracellular antigen‐binding domain, usually an scFv that determines antigen specificity and affinity; a hinge region that provides spatial flexibility; a transmembrane domain that anchors the receptor to the cell membrane; and an intracellular signaling domain that converts antigen recognition into effector‐cell activation [13, 14] This modular design enables independent optimization of target recognition, receptor geometry, membrane stability, and downstream signaling, thereby supporting the adaptation of CAR technology beyond conventional CAR‐T cells.

CAR technology is commonly described as having evolved through five structural generations (Figure 1). First‐generation CARs contain only CD3ζ as the activation domain and show limited expansion and persistence. Second‐generation CARs incorporate a single costimulatory domain, most commonly CD28 or 4‐1BB, and form the backbone of most clinically approved CAR‐T products. Third‐generation CARs combine two costimulatory domains, such as CD28 with 4‐1BB or OX40, although their clinical superiority over second‐generation designs remains variable [15]. Fourth‐generation CARs, also known as armored CARs or TRUCKs, are engineered to release cytokines, chemokines, checkpoint‐blocking molecules, or other functional mediators upon antigen recognition, thereby enabling active remodeling of tumor, inflammatory, or fibrotic microenvironments. Fifth‐generation CARs further incorporate cytokine receptor‐derived signaling modules, such as IL‐2Rβ/STAT3/STAT5 motifs, to integrate antigen‐dependent activation, costimulation, and JAK–STAT‐mediated cytokine‐like signaling [16]. Although these designs aim to enhance expansion, survival, and functional persistence, their long‐term safety, signaling controllability, and manufacturing complexity still require systematic evaluation (Table 1).

**FIGURE 1:**
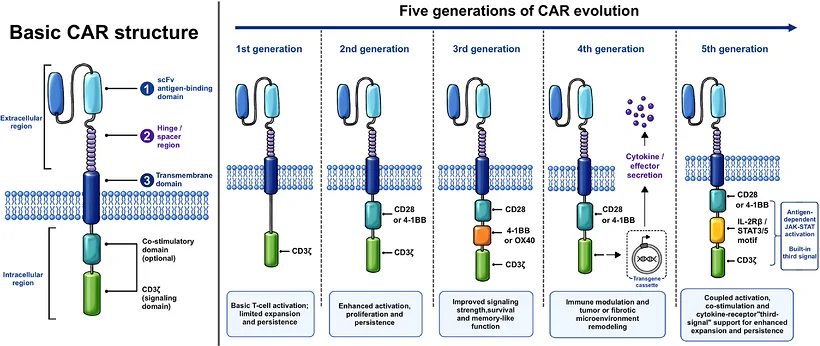
Schematic overview of the basic CAR structure and the structural evolution of five generations of CAR design.

**TABLE 1:** Comparison of Structural Design Features Across Three CAR Platforms.

Design dimension	CAR‐T	CAR‐M	CAR‐NK
Antigen‐recognition domain	Predominantly scFv‐based, typically composed of VH‐linker‐VL; nanobodies and receptor/ligand‐derived binding domains may also be used.	Predominantly scFv‐based, but antibody fragments, receptor/ligand‐derived binding domains, or phagocytosis‐related recognition modules may also be incorporated.	Predominantly scFv‐based, but the extracellular region of NKG2D, nanobodies, or other NK‐adapted recognition structures may also be used.
Hinge/spacer region	CD8α, CD28, IgG4‐Fc spacer, and related designs.	CD8α, CD28, IgG4‐Fc spacer, and related designs, with length optimized according to phagocytic synapse formation.	CD8α, CD28, IgG4‐Fc spacer, and related designs, with attention to both CAR expression and NK immune synapse formation.
Transmembrane domain	CD8α, CD28, and related designs.	CD8α, CD28, or transmembrane regions derived from specific functional receptors.	CD8α, CD28, NKG2D, and other NK‐adapted transmembrane domains.
Major intracellular signaling domains	CD3ζ plus CD28 or 4‐1BB; fifth‐generation CARs may further incorporate IL‐2Rβ/STAT3/5 and related JAK‐STAT signaling modules.	FcRγ, Megf10, MerTK, Bai1, CD3ζ, TLR/TIR, CD147, and related modules.	CD3ζ, 2B4, DAP10, DAP12, 4‐1BB, and NKG2D‐associated signaling modules.
Platform‐specific functional modules	Armored cytokines such as IL‐12, IL‐15, and IL‐18; chemokines; immune checkpoint‐blocking molecules; fifth‐generation JAK‐STAT “signal 3” modules; safety switches.	TLR/TIR to promote M1‐like polarization; FcRγ, Megf10, or MerTK to enhance phagocytosis; CD147 to promote MMP‐associated ECM remodeling; IFN‐γ‐related modules to strengthen inflammatory reprogramming.	IL‐15 or membrane‐bound IL‐15 to enhance persistence; 2B4, DAP10, or DAP12 to enhance NK activation; high‐affinity CD16 configurations to strengthen ADCC; safety switches or chemokine receptors to enhance controllability and homing.
Engineering/delivery strategies	Primarily lentiviral and retroviral systems; mRNA electroporation, transposon systems, and T‐cell‐targeted LNP approaches are emerging strategies.	Adenoviral or lentiviral systems, mRNA/LNP delivery, nanoparticle‐based in vivo reprogramming, and iPSC‐derived macrophage engineering.	Lentiviral or retroviral systems, mRNA electroporation, transposon systems, and iPSC‐NK engineering platforms.
Design priorities	To enhance antigen‐specific cytotoxicity, in vivo expansion, persistence, and immunological memory, while controlling CRS, ICANS, and related immune toxicities.	To emphasize phagocytic clearance, antigen presentation, remodeling of inflammatory/fibrotic microenvironments, and ECM degradation.	To emphasize CAR‐dependent killing, cooperation with natural NK receptors, ADCC, safety, and off‐the‐shelf manufacturing potential.

The intracellular signaling logic of CARs must be adapted to the biology of each effector‐cell platform. CAR‐T cells mainly rely on TCR‐derived signaling modules, and the combination of CD3ζ with CD28 or 4‐1BB has been clinically validated for more than a decade. In contrast, CAR‐M cells require macrophage‐intrinsic signaling domains because macrophages do not express the TCR complex [17]. Foundational screening studies showed that phagocytic receptor‐derived domains such as Megf10 and FcRγ can confer strong antigen‐dependent phagocytosis, whereas MerTK and Bai1 show more limited effects [18]. Subsequent CAR‐M designs have expanded this toolkit by incorporating modules that promote inflammatory polarization, ECM remodeling, or whole‐cell phagocytosis, including TLR4/TIR, IFN‐γ receptor‐derived domains, CD147‐associated domains, and PI3K‐recruiting motifs. CAR‐NK cells share some structural features with CAR‐T cells but often incorporate NK‐adapted signaling modules, such as DAP10, DAP12, 2B4, or NKG2D‐derived elements, to better align CAR signaling with endogenous NK‐cell activation, degranulation, and cytokine‐production pathways [19]. Thus, CAR‐T, CAR‐M, and CAR‐NK platforms differ not only in effector‐cell identity but also in the intracellular signaling architecture required to match their native biology.

### 2.2 Target Landscape of Fibrosis and Inflammation

The selection of target antigens is a central challenge for all CAR‐based therapies. An ideal target antigen should be highly and uniformly expressed on pathogenic cells or within pathological microenvironments, while being absent or expressed at low levels in critical normal tissues. However, targets in fibrotic and inflammatory diseases often fail to meet this ideal requirement and instead present a unique “specificity paradox.”

To systematically understand CAR targets in fibrotic and inflammatory diseases, they can be classified along three dimensions (Figure 2): pathological attributes, expression specificity, and expression dynamics. First, according to their pathological attributes, current targets can be broadly divided into three categories: pathogenic immune‐cell targets, pathogenic stromal‐cell targets, and microenvironment‐associated molecular targets. CD19 and BCMA represent B‐cell lineage and plasma‐cell targets, respectively, and are mainly applicable to autoimmune diseases driven by autoantibodies or aberrant B‐cell responses [20, 21, 22]. FAP, uPAR, PDGFRβ, TNC, and DPP4 primarily mark activated fibroblasts, hepatic stellate cells, ECM‐producing cell populations, or specific pathological stromal regions, making them major candidate targets for anti‐fibrotic CAR therapies [23, 24, 25, 26]. In contrast, microenvironment‐associated molecules such as TNF, CD47, and OxLDL reflect design strategies centered more on signal modulation or phagocytosis enhancement rather than conventional target‐cell killing [27].

**FIGURE 2:**
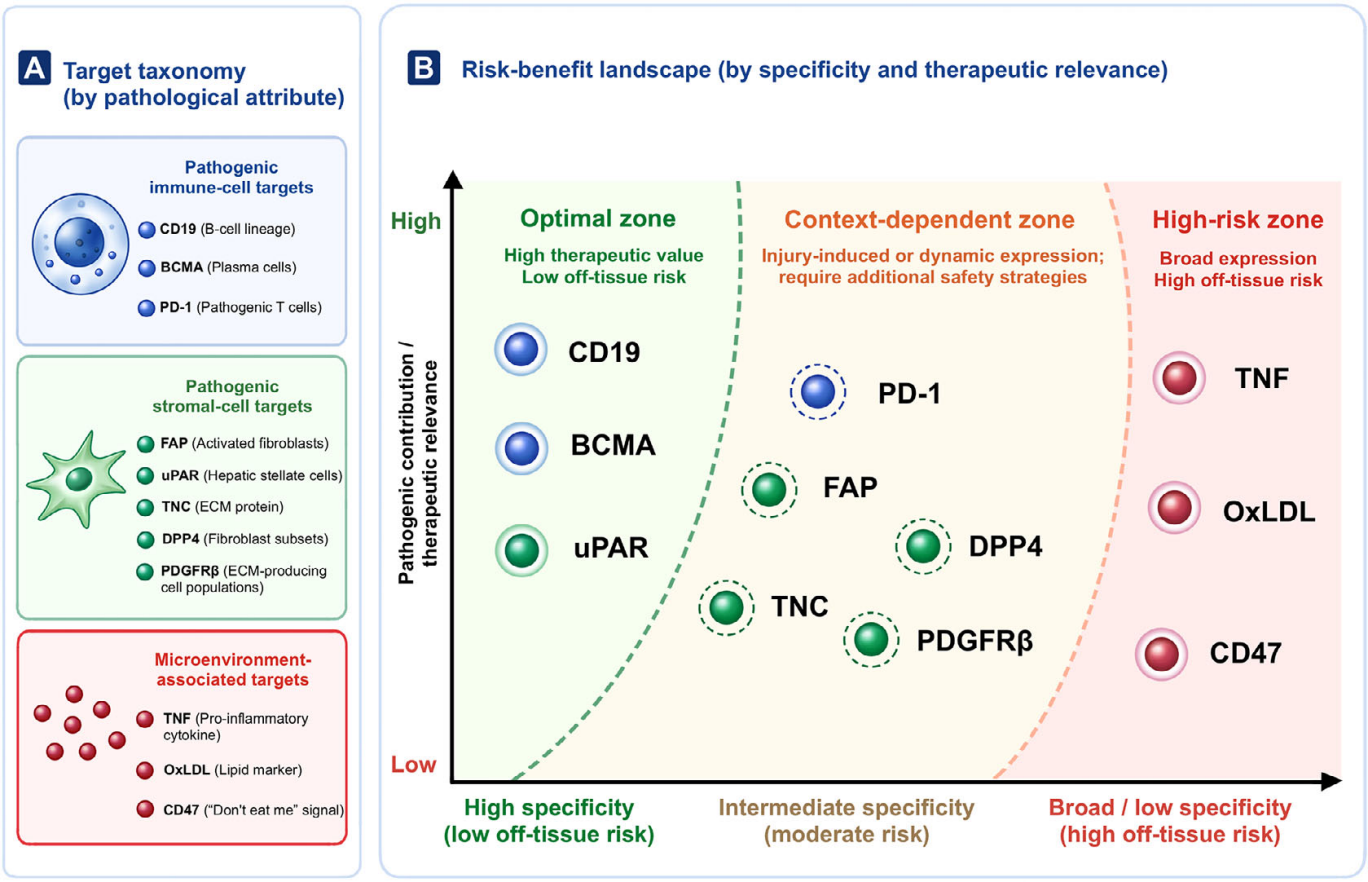
Target taxonomy and risk–benefit landscape of CAR targets in fibrotic and inflammatory diseases. This schematic summarizes representative CAR targets according to their pathological attributes and therapeutic relevance in non‐oncological disease settings. (A) Candidate targets are classified into three major categories: pathogenic immune‐cell targets, including CD19, BCMA, and PD‐1; pathogenic stromal‐cell targets, including FAP, uPAR, TNC, DPP4, and PDGFRβ; and microenvironment‐associated targets, including TNF, OxLDL, and CD47. (B) The risk–benefit landscape positions these targets according to their disease relevance and tissue specificity. Highly disease‐relevant and specific targets are located in the optimal zone, whereas dynamically expressed stromal targets fall into a context‐dependent zone requiring additional safety control. Broadly expressed microenvironment‐associated targets are placed in the high‐risk zone because of their greater off‐tissue toxicity potential.

Second, CAR targets differ substantially in their expression specificity and temporal dynamics, which directly determine platform selection and safety‐control strategies. Highly specific targets such as CD19 and BCMA are largely restricted to defined immune‐cell lineages and are therefore suitable for durable CAR‐T‐mediated deep immune clearance. Moderately specific targets such as FAP, uPAR, and PDGFRβ are highly expressed in pathological cells but may also show background expression in certain normal repair‐associated cells or tissues. These targets therefore require additional safety strategies, including affinity tuning, logic gating, transient expression, or local delivery, to reduce on‐target off‐tissue toxicity. Broadly expressed targets such as TNF and CD47 are generally unsuitable for simple cytotoxic CAR designs but may be exploited through microenvironment‐sensing switches or phagocytosis‐modulating modules to create a therapeutic window. Meanwhile, injury‐induced targets such as FAP and uPAR display marked temporal dependence, with expression levels changing according to tissue injury, inflammatory activity, and fibrotic stage. This suggests that anti‐fibrotic CAR therapy should not only select appropriate targets but also match the optimal delivery window and duration of CAR expression. Based on this rationale, mRNA‐based transient CARs, locally delivered CARs, and controllable switch‐based CARs may be more suitable than conventional long‐persisting CARs for certain fibrotic and chronic inflammatory settings (Table 2).

**TABLE 2:** Representative CAR Target Landscape.

Target	Target source / target cell or pathological structure	Applicable platform	Disease area	Translational stage	Notes
CD19	pre‐B to mature B cells, memory B cells, and a subset of plasmablasts	CAR‐T; CAR‐NK under exploration	SLE, SSc, RA, MS, myositis, and related disorders	FDA‐approved; Phase I/IIa in autoimmune diseases	The most mature B‐cell reset target in autoimmune disease
BCMA	Plasma cells / long‐lived plasma cells	CAR‐T; CAR‐NK under exploration	SLE/LN and antibody‐mediated autoimmune diseases	FDA‐approved; Phase I/IIa in autoimmune diseases	Complements CD19 by targeting plasma cells
FAP	Activated fibroblasts, myofibroblasts, and activated hepatic stellate cells (aHSCs)	CAR‐T, CAR‐M, CAR‐NK	Cardiac, hepatic, pulmonary, renal, and related fibrotic diseases	Preclinical	Prototypical antifibrotic CAR target; off‐tissue risk
uPAR	Senescent cells, activated HSCs, and pathological stromal cells	CAR‐T; CAR‐M under exploration	Liver fibrosis and senescence‐related disorders	Preclinical	More precisely categorized as one of the senolytic CAR targets
PDGFRβ	PDGFRβ+ ECM‐producing cells, including pericytes, myofibroblasts, and related populations	CAR‐T	Renal fibrosis / CKD	Preclinical	Pericyte‐ and vasculature‐related safety requires careful evaluation
TNC	Pathological ECM component / fibrotic matrix	CAR‐M	Liver fibrosis and related conditions	Preclinical	Represents an ECM‐targeting strategy rather than a typical cell‐surface target
DPP4/CD26	DPP4+ fibroblasts / stromal cells associated with cutaneous fibrosis	CAR‐M under exploration	Cutaneous fibrosis and scar‐related disorders	Preclinical	Because DPP4 expression is relatively broad, selectivity and local safety should be emphasized
TNF	TNF‐high inflammatory microenvironment / soluble TNF signaling	CAR‐M signal‐switch platform	Chronic inflammation and renal/hepatic injury models	Preclinical	Inflammation‐responsive CAR‐M signal‐switch strategy
CD47	CD47‐high apoptotic cells, foam cells, and diseased cells	CAR‐M	Atherosclerosis	Preclinical	Promotes efferocytosis by relieving the 'don't eat me' signal
OxLDL	Oxidized lipid / atherosclerotic plaque‐associated antigen	CAR‐Treg	Atherosclerosis	Preclinical	Represents an immunoregulatory CAR‐Treg strategy rather than a cytotoxic CAR‐T approach

## 3 CAR‐T Cell Therapy From Systemic Autoimmunity to Multiorgan Fibrosis

### 3.1 Mechanisms and Principles of CAR‐T Cell Therapy

The relatively mature CAR‐T workflow provides the technical basis for extending this platform beyond oncology. Classical autologous production comprises T‐cell collection, activation, CAR transduction, expansion, lymphodepletion and reinfusion [28]. However, translation to autoimmune diseases and fibrosis still depends on disease‐specific optimization of CAR design, persistence, safety and accessibility [29].

Among these, the choice of costimulatory domain and manufacturing model are two central factors shaping the therapeutic characteristics of CAR‐T cells. CD28 costimulation generally confers faster expansion and stronger acute effector function, but may be associated with shorter persistence and higher inflammatory toxicity. In contrast, 4‐1BB costimulation tends to promote a memory‐like phenotype and longer‐term persistence, and may be associated with a relatively milder cytokine‐release profile; accordingly, it is more commonly used in CAR‐T studies for autoimmune diseases [30]. In terms of manufacturing models, autologous CAR‐T cells offer advantages in personalization and immune compatibility, but their high cost and long production time limit broader application in chronic non‐oncological diseases [31]. Meanwhile, lipid nanoparticle mRNA‐mediated in vivo CAR‐T generation aims to bypass ex vivo manufacturing altogether, offering a more flexible “factory‐free” therapeutic route for diseases such as fibrosis that may require short‐term and controllable intervention.

### 3.2 Clinical Applications of CAR‐T Cell Therapy in Autoimmune and Inflammatory Diseases

#### 3.2.1 Systemic Lupus Erythematosus

Systemic lupus erythematosus (SLE) is the best‐supported non‐oncological indication for CAR‐T therapy. Its B‐cell‐driven autoantibody pathology and incomplete control by conventional immunosuppression provide a clear rationale for deep B‐cell depletion in refractory disease [32].

In 2021, the Schett group reported the first case of CD19 CAR‐T therapy for SLE, marking the emergence of this field [33]. In 2022, the same group published complete treatment data for five patients with refractory SLE, including four with lupus nephritis, in Nature Medicine. Across the five patients, treatment induced rapid B‐cell depletion, remission according to the DORIS criteria, and withdrawal of immunosuppressive therapy. The subsequent reconstitution of a predominantly naïve B‐cell repertoire was consistent with an “immune reset” rather than permanent B‐cell aplasia [34].

In 2024, the same group expanded the cohort to 15 patients, including 8 with SLE, 4 with systemic sclerosis, and 3 with myositis, further supporting the value of early intervention. Patients with shorter disease duration (<5 years) showed a higher complete remission rate than those with longer disease duration (>5 years), suggesting that earlier use of CAR‐T therapy before irreversible organ damage occurs may achieve more effective immune resetting. After B‐cell reconstitution, all patients showed a naïve B‐cell phenotype and disappearance of autoreactive clones [35].

A 2024 phase I study evaluated compound CD19/BCMA CAR‐T cells in 13 patients with SLE, 12 of whom received the standard dose. All standard‐dose patients achieved medication‐free remission, with autoantibody clearance, complement normalization and renal improvement; only grade ≤2 CRS and no ICANS were reported. These findings suggest deeper depletion extending to long‐lived plasma cells, but require confirmation in larger controlled cohorts [36].

#### 3.2.2 Systemic Sclerosis and Inflammatory Myopathies

Systemic sclerosis (SSc) represents a more complex challenge for CAR‐T therapy, as it involves both B‐cell‐driven autoimmunity and extensive cutaneous and visceral fibrosis. In 2023, the Schett group reported preliminary results from three patients with SSc treated with CD19 CAR‐T cells. After treatment, the modified Rodnan skin score (mRSS) decreased by an average of 8.3 points, pulmonary function remained stable or improved, autoantibody levels declined markedly, and no severe adverse events were observed. CAR‐T therapy may interrupt the connection between immune activation and fibrotic progression by eliminating B cells that produce pro‐fibrotic mediators, thereby creating a permissive environment for tissue repair [37].

TyU19 is a multiplex‐edited allogeneic CAR‐T product designed to reduce alloreactivity and exhaustion [38] (Figure 3A). In three patients with severe autoimmune diseases, it induced rapid B‐cell depletion and early clinical improvement, with no GVHD or CRS reported during follow‐up. However, the very small cohort and short observation period preclude conclusions regarding durability and long‐term safety.

**FIGURE 3:**
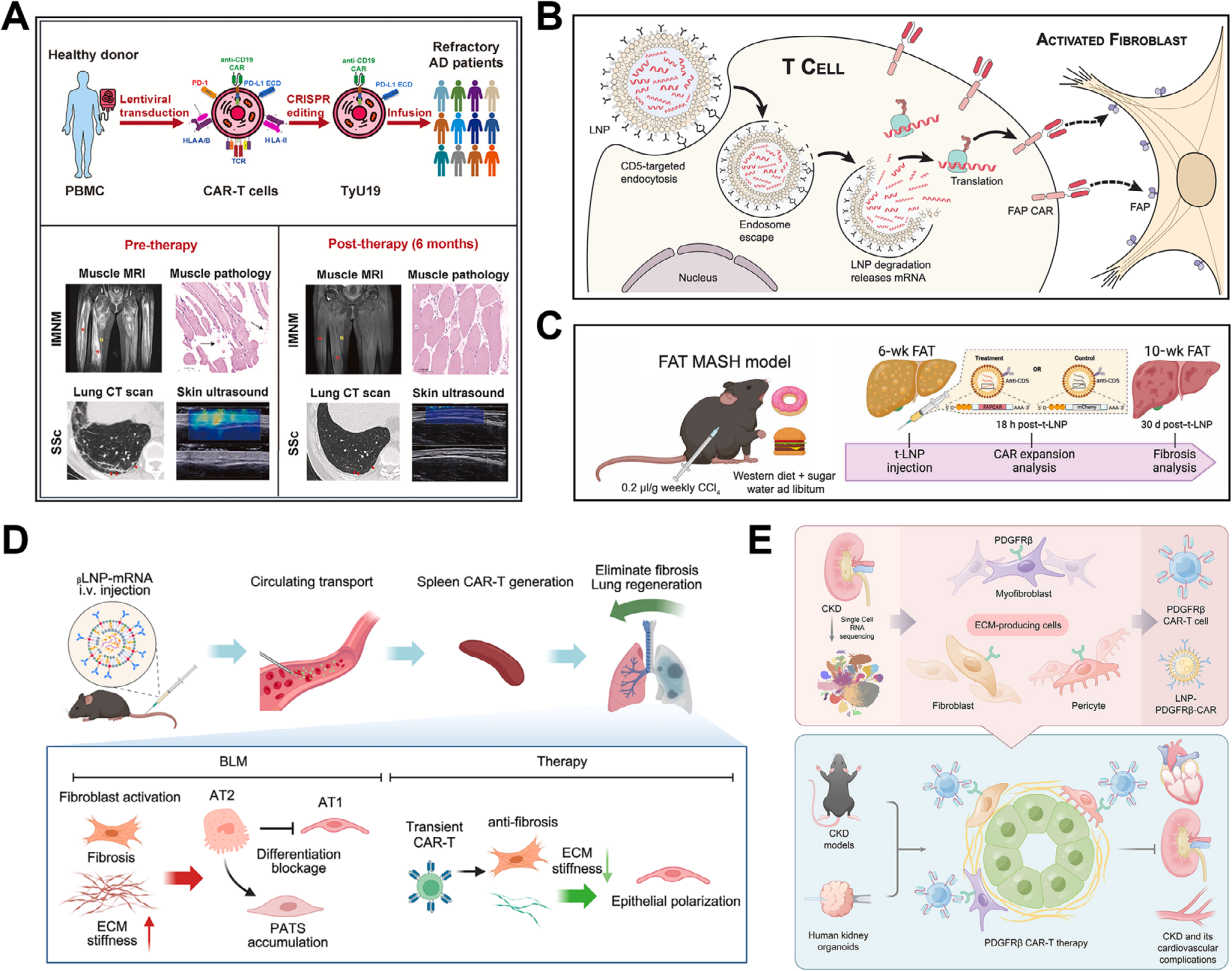
Schematic illustration of CAR‐T cell applications in fibrotic diseases. (A) Schematic illustration of allogeneic CD19‐targeted CAR‐T cell therapy in patients with severe myositis and systemic sclerosis. Reproduced with permission [38]. Copyright Year 2024. Cell Press. (B) Schematic outlining the molecular process to create transient FAPCAR T cells using CD5‐targeted LNPs. Reproduced with permission [43]. Copyright Year 2022. American Association for the Advancement of Science. (C) Schematic illustration of in vivo‐generated anti‐FAP CAR‐T cells for reducing fibrosis and restoring liver homeostasis in metabolic dysfunction–associated steatohepatitis. Reproduced with permission [45]. Copyright Year 2026. American Association for the Advancement of Science. (D) Schematic illustration of LNP‐mRNA–mediated in vivo generation of transient anti‐FAP CAR‐T cells for eliminating overactivated fibroblasts, reducing pulmonary fibrosis, and promoting alveolar epithelial regeneration. Reproduced with permission [46]. Copyright Year 2025. Springer Nature. (E) Schematic illustration of PDGFRβ‐targeted CAR‐T cell therapy for eliminating ECM‐producing cells and alleviating fibrosis in chronic kidney disease. Reproduced with permission [47]. Copyright Year 2025. Cell Press.

#### 3.2.3 Rheumatoid Arthritis and Multiple Sclerosis

In rheumatoid arthritis (RA), Freeley summarized clinical data from 10 patients with RA who had received CD19/BCMA or CD20–CD19 tandem CAR‐T therapy by the end of 2024. Nine of the ten patients with seropositive RA showed marked clinical responses, corresponding to a 90% medication‐free remission rate. These responses included depletion of circulating B cells, reduction or even seroconversion of anti‐citrullinated protein antibodies (ACPAs), significant improvement in DAS28‐CRP scores, and achievement of medication‐free remission. Notably, the tenth patient, who had seronegative RA, initially responded but subsequently relapsed, highlighting the heterogeneity of RA. This observation suggests that certain RA subgroups may be driven more predominantly by T‐cell‐mediated mechanisms rather than B‐cell‐dependent pathology, and that B‐cell depletion alone may be insufficient in these patients [39].

In multiple sclerosis (MS), KYV‐101, a CD19‐targeted CAR‐T product incorporating a 4‐1BB costimulatory domain, has demonstrated the ability to penetrate the blood–brain barrier in patients with progressive MS. CAR‐T cells were able to enter the central nervous system and eliminate perivascular B‐cell aggregates, leading to an approximately 75% reduction in cerebrospinal fluid oligoclonal bands and preventing the emergence of gadolinium‐enhancing lesions in approximately 85% of patients. Preliminary efficacy of CD19 CAR‐T therapy has also been reported in other B‐cell‐mediated autoimmune diseases, including myasthenia gravis, neuromyelitis optica spectrum disorder, and immune thrombocytopenia, although current evidence remains limited to individual case reports or early clinical observations [40].

#### 3.2.4 Next‐Generation CAR Strategies for Autoimmune Diseases

Although CD19‐directed CAR‐T cells currently have the strongest clinical evidence in autoimmune diseases, they may incompletely eliminate long‐lived plasma cells. BCMA‐directed CAR‐T cells could deepen depletion in plasma‐cell‐driven disorders, but they may leave earlier autoreactive B‐cell compartments insufficiently targeted while also removing protective plasma cells, thereby increasing the risks of hypogammaglobulinaemia and infection. Dual CD19/BCMA targeting extends coverage across multiple stages of B‐cell differentiation and may enable a more comprehensive immune reset. However, the resulting depth and duration of humoral immune suppression may be excessive for non‐malignant diseases [41].

By contrast, chimeric autoantibody receptor T cells (CAAR‐T cells) display a disease‐associated autoantigen to selectively recognize B‐cell receptors expressed by autoreactive clones, potentially preserving unrelated B‐cell populations. This approach is particularly attractive for diseases dominated by a well‐defined autoantigen, such as pemphigus vulgaris, but its applicability may be limited by antigen and epitope heterogeneity, epitope spreading, and interference from circulating autoantibodies. CAR‐Treg cells follow a different therapeutic logic by promoting antigen‐directed immune tolerance rather than cytotoxic depletion. Their successful translation will depend on stable FOXP3 expression, durable suppressive function, appropriate tissue trafficking, and the identification of sufficiently restricted target antigens [42].

In vivo CAR engineering may further improve accessibility by generating CAR‐expressing immune cells directly within patients. Transient mRNA‐based approaches may provide time‐limited activity, whereas integrating or persistently expressed systems could offer longer‐lasting effects. Nevertheless, in vivo engineering provides less control over cellular phenotype, biodistribution, transduction efficiency, and dose than conventional ex vivo manufacturing and may require repeat administration when expression is transient.

These strategies should therefore be viewed as functionally complementary rather than as successive generations of a single treatment pathway. BCMA‐directed and dual‐target CARs prioritize depletion depth, CAAR‐T cells prioritize autoreactive‐clone specificity, CAR‐Tregs aim to restore immune tolerance, and in vivo engineering primarily addresses manufacturing and treatment accessibility. Clinical selection should be guided by the dominant pathogenic cell population, the degree of antigen specificity, the required duration of therapeutic activity, and the acceptable depth of immune depletion.

### 3.3 CAR‐T Cell Therapy in Fibrotic Diseases

In fibrosis, CAR‐T development has increasingly shifted toward transient in vivo programming to limit persistence during dynamic tissue repair.

#### 3.3.1 Cardiac Fibrosis: A Proof‐of‐Concept Study of In Vivo CAR‐T Therapy

Rurik et al. used CD5‐targeted LNPs to transiently program endogenous T cells to express a FAP CAR in vivo [43] (Figure 3B). In a murine cardiac fibrosis model, a single dose reduced fibrosis and improved cardiac function, while CAR expression largely subsided within 1 week. This study provided preclinical proof of concept for a non‐integrating, time‐limited in vivo CAR‐T strategy, although its efficacy and safety in humans remain untested.

Du et al., in their study of in vivo CAR‐M therapy, also analyzed the cellular composition of cardiac fibrosis. They found that FAP^+^ fibroblasts accounted for only a subset of activated stromal cells and were less abundant than the total αSMA/PDGFRα double‐positive cell population. This suggests that a single‐target FAP strategy may provide incomplete cellular coverage in cardiac fibrosis, thereby supporting the design of dual‐target strategies, such as FAP plus PDGFRα, or broader targeting approaches [44].

#### 3.3.2 Liver Fibrosis

In MASH models, CD5‐targeted LNPs generated transient FAP CAR‐T cells in vivo, reducing the burden of activated hepatic stellate cells, collagen deposition, and fibrosis while improving metabolic indices [45] (Figure 3C). These findings demonstrate preclinical efficacy, but their relevance to human MASH remains to be established.

#### 3.3.3 Lung Fibrosis

In a bleomycin‐induced pulmonary fibrosis model, an optimized CD5‐targeted LNP induced transient FAP CAR expression in endogenous T cells and reduced fibrosis [46] (Figure 3D). Single‐cell analyses linked fibroblast depletion and matrix normalization to reparative programmes in epithelial cells and macrophages, although these associations remain correlative and have not yet been validated in humans.

#### 3.3.4 Kidney Fibrosis

PDGFRβ‐directed CAR‐T cells were evaluated across four murine CKD models using both ex vivo–engineered products and CD5‐targeted LNPs for in vivo CAR generation [47] (Figure 3E). Both approaches reduced renal fibrosis and improved measures of kidney function, with additional validation in human kidney organoids. The broader stromal distribution of PDGFRβ may enhance therapeutic coverage, but it also increases the need to assess potential on‐target effects on normal mesangial cells, pericytes, and other perivascular stromal populations.

#### 3.3.5 CAR‐Treg Subsets: A Dual‐Function Strategy Combining Cytotoxicity and Immune Regulation

CAR‐Tregs provide a distinct strategy that combines antigen‐directed localization with immune regulation rather than relying solely on conventional cytotoxicity. In preclinical pulmonary‐fibrosis and atherosclerosis models, FAP1‐ or OxLDL‐directed CAR‐Treg products reduced pathological fibroblast or inflammatory activity [48, 49]. However, lineage stability and the stage‐dependent effects of Treg‐associated TGF‐β programmes require careful temporal and safety evaluation. CAR‐T cells have also reduced hepatic injury and fibrosis by eliminating PD‐1‐high pathogenic tissue‐resident T cells in a preclinical autoimmune‐cholangitis model, extending targeting beyond B‐lineage cells [50].

Collectively, fibrosis studies indicate a shift toward transient in vivo CAR programming, which may better align therapeutic activity with the time‐limited requirements of tissue repair [51].

## 4 Macrophage‐Based CAR Therapy for Non‐Cancer Applications

The rationale for using macrophages as CAR effector‐cell carriers lies in their intrinsic involvement in tissue inflammation, repair, and fibrosis. Unlike CAR‐T cells, which mainly rely on cytotoxic killing, CAR‐M cells can combine antigen‐dependent target recognition with tissue infiltration, phagocytic clearance, matrix remodeling, antigen presentation, and local immune modulation. Resident macrophages, such as Kupffer cells, alveolar macrophages, cardiac macrophages, and renal interstitial macrophages, participate in organ homeostasis and injury repair, whereas circulating monocytes can be recruited to lesions through chemokine axes such as CCR2–CCL2 and differentiate into inflammation‐associated macrophages. This tissue localization and lesion‐homing capacity provides CAR‐M cells with distinct advantages in local fibrotic and chronic inflammatory microenvironments [52, 53, 54].

CAR‐M engineering can tune phagocytosis, matrix degradation, antigen presentation and inflammatory state through macrophage‐adapted signaling domains. Because polarization is context dependent, the aim should be controllable, disease‐stage‐specific programming rather than fixed M1‐ or M2‐like activation [55, 56].

### 4.1 CAR Structural Design in CAR‐M Cells

The structural design of CAR‐M is far more complex than that of CAR‐T, because it involves not only “activation” and “targeting,” but also the selection of appropriate phagocytic signaling modules and the induction of specific macrophage polarization states.

#### 4.1.1 Screening and Comparison of Phagocytic Signaling Domains

Foundational screening identified Megf10 and FcRγ as strong phagocytic signaling domains, with CD3ζ showing intermediate activity and MerTK or Bai1 weaker effects [18]. Megf10 may favor relatively restrained clearance, whereas FcRγ couples phagocytosis to stronger inflammatory signaling; tandem ITAM–PI3K designs can further promote whole‐cell engulfment [57]. Domain selection should therefore match the desired balance between clearance and inflammation.

#### 4.1.2 Polarization and Functional Regulation Domains

CAR‐M signaling can be tailored beyond phagocytosis: TLR4/TIR or IFN‐γR modules promote inflammatory activation, CD147‐associated modules enhance MMP‐dependent matrix remodeling, and MERTK‐oriented designs support efferocytosis and resolution [58, 59]. These functions should be matched to disease stage rather than used to lock macrophages into a fixed polarization state.

#### 4.1.3 Signal‐Gated CARs: From “Recognition–Killing” to “Sensing–Reprogramming”

A TNF‐responsive CAR‐M design replaces a conventional killing signal with IL‐4Rα signaling, converting a pro‐inflammatory cue into a regulatory programme [60]. This sensing–reprogramming logic may preserve target recognition without broad TNF blockade, although its long‐term effects may vary with disease stage and fibrotic context.

### 4.2 Applications of CAR‐M Cell Therapy in Fibrotic Diseases

#### 4.2.1 Liver Fibrosis

The liver is a compelling CAR‐M setting because macrophages naturally traffic to injured tissue and can influence both hepatic stellate‐cell clearance and matrix resolution [61].uPAR‐directed CAR‐M cells accumulated in fibrotic livers, cleared activated hepatic stellate cells and reduced fibrosis across three murine injury models [62] (Figure 4A). The study also suggested that antigen presentation after phagocytosis may extend the response beyond the short persistence of transferred macrophages. These findings remain preclinical and require validation of human uPAR distribution and target safety.

**FIGURE 4:**
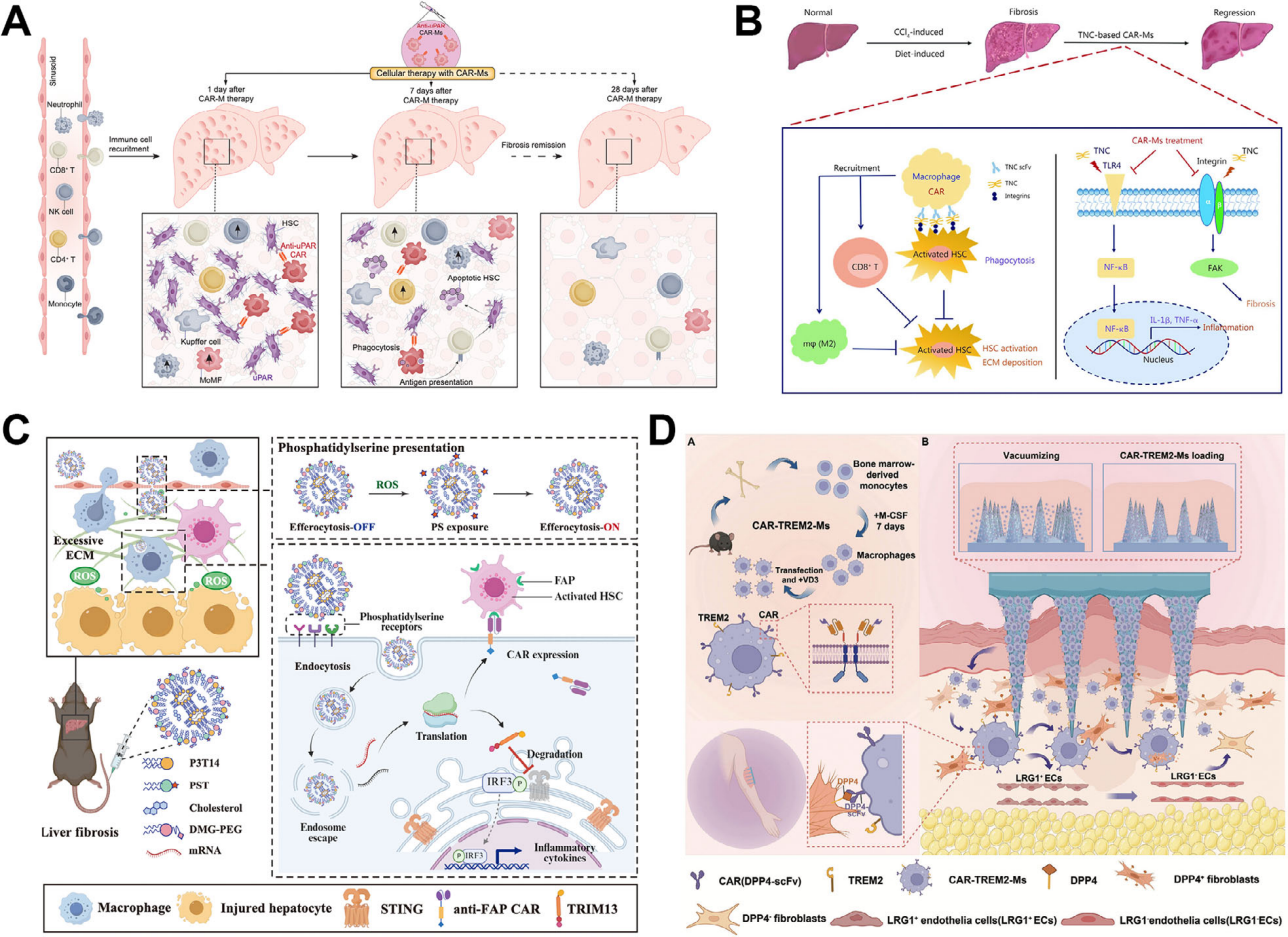
Schematic illustration of CAR‐M cell applications in fibrotic diseases. (A) Schematic illustration of uPAR‐targeted CAR‐M therapy for liver fibrosis and cirrhosis, in which CAR‐engineered macrophages exert direct antifibrotic effects against hepatic stellate cells and promote specific T‐cell responses. Reproduced with permission [62]. Copyright Year 2024. Elsevier. (B) Schematic illustration of TNC‐targeted CAR‐M therapy for liver fibrosis, in which CAR‐engineered macrophages recognize TNC‐expressing hepatic stellate cells, promote their phagocytic clearance, reduce ECM deposition, and modulate the liver immune microenvironment. Reproduced with permission [63]. Copyright Year 2025. BioMed Central. (C) Schematic illustration of ESLNP‐mediated in vivo engineering of macrophages with anti‐FAP CAR and TRIM13 in ROS‐enriched fibrotic liver, enabling activated hepatic stellate cell‐specific clearance, inflammatory resolution, and reversal of liver fibrosis. Reproduced with permission [64]. Copyright Year 2026. Springer Nature. (D) S Schematic illustration of CAR‐TREM2 macrophages engineered with a DPP4‐scFv CAR and delivered by microporous microneedles to phagocytose DPP4‐positive fibroblasts, regulate endothelial cell fibrotic phenotypes, and promote scarless wound regeneration. Reproduced with permission [68]. Copyright Year 2024. Wiley‐Blackwell.

TNC‐directed CAR‐M cells extend targeting from activated cells to a spatially restricted fibrotic‐matrix component [63] (Figure 4B), in a murine liver‐fibrosis model, this approach reduced TNC‐associated signaling and fibrosis, suggesting that matrix anchoring may improve lesion localization. Its safety will depend on distinguishing pathological matrix from physiological repair.

Gao et al. further proposed a TRIM13‐enhanced in situ CAR‐M engineering strategy, providing a dual‐functional paradigm of “targeted phagocytosis plus inflammation resolution” for CAR‐M‐based fibrosis therapy [64] (Figure 4C). This ROS‐responsive ESLNP system delivered anti‐FAP CAR and TRIM13 mRNAs to lesion‐associated macrophages, generating transient anti‐inflammatory CAR‐M cells in fibrotic liver. The CAR promoted phagocytosis of FAP^+^ activated HSCs, while TRIM13 suppressed STING‐dependent inflammation and favored a Ly6C^lo/CD206^+^ phenotype. In CCl_4_‐ and HFMCD‐induced fibrosis, this strategy reduced liver injury and matrix deposition, highlighting the importance of combining pathological‐cell clearance with sustained anti‐inflammatory CAR‐M programming.

#### 4.2.2 Cardiac Fibrosis

Megf10‐based FAP CAR‐M cells infiltrated fibrotic myocardium, reduced activated fibroblasts and collagen deposition, and improved cardiac function in an AngII/PE model [65]. Short‐term assessments showed limited systemic inflammatory toxicity, supporting restrained phagocytic signaling as a potential advantage, although longer safety evaluation is required. CD147‐based FAP CAR‐M cells reduced fibroblast burden and fibrosis after myocardial ischemia–reperfusion injury while improving cardiac function [66]. The design may couple target clearance to MMP‐associated matrix remodeling; reported metabolic normalization was correlative, and safety assessment remained short term.

Liu et al. reported a Legumain‐based dual‐mRNA strategy in *Advanced Materials* in 2025 [67], addressing a critical functional bottleneck of CAR‐M therapy in cardiac fibrosis. In a murine myocardial‐infarction model, it reduced fibrosis and improved function, illustrating the potential value of engineering macrophage intracellular processing as well as antigen recognition.

In vivo generation of FAP CAR‐M, as reported in Circulation Research in 2025 [44] (Figure 5A), further simplified the manufacturing challenge of macrophage‐based platforms. This strategy used DOPS‐modified LNPs encapsulating FAP CAR mRNA. DOPS, or phosphatidylserine, functions as an “eat‐me” signal on apoptotic cells and is naturally recognized by macrophages. After intravenous injection, DOPS‐LNPs were preferentially taken up by endogenous macrophages, enabling transient in situ expression of FAP CAR without any ex vivo cell manipulation.

**FIGURE 5:**
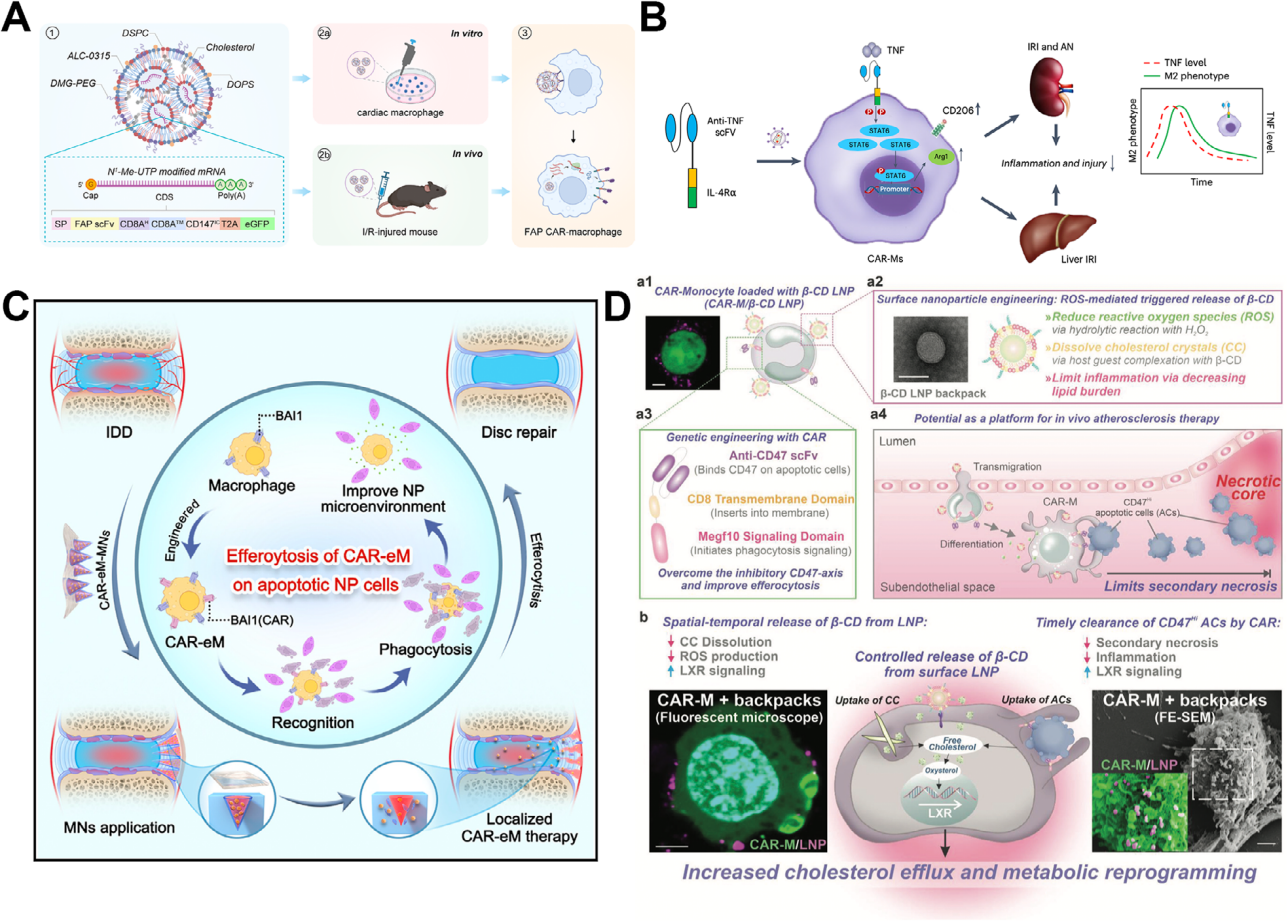
Schematic illustration of CAR‐M therapy in fibrotic and inflammatory diseases. (A) Schematic illustration of LNP‐mRNA–mediated generation of FAP CAR macrophages in vitro and in vivo. LNPs encapsulating modified mRNA encoding a FAP CAR construct are delivered to macrophages either ex vivo or by intravenous injection in myocardial I/R‐injured mice, enabling transient expression of FAP CAR and functional programming of macrophages. Reproduced with permission [44]. Copyright Year 2025. Lippincott Williams and Wilkins Ltd. (B) Schematic illustration of anti‐TNF/IL‐4Rα CAR‐Ms for inflammation‐responsive immunotherapy. These engineered macrophages capture TNF through an anti‐TNF scFv and convert inflammatory TNF signals into IL‐4Rα‐mediated anti‐inflammatory signaling, inducing a reversible M2‐like phenotype to reduce inflammation and tissue injury in kidney and liver inflammatory disease models. Reproduced with permission [60]. Copyright Year 2025. Springer Nature. (C) Schematic illustration of microneedle‐mediated delivery of CAR‐M‐like engineered macrophages targeting efferocytosis to enhance phagocytic clearance and treat inflammatory intervertebral disc degeneration. Reproduced with permission [69]. Copyright Year 2025. Elsevier. (D) Schematic illustration of nano‐immunoengineered anti‐CD47 CAR‐Ms for potential atherosclerosis therapy. β‐CD LNP‐loaded CAR‐Ms home to inflamed atherosclerotic lesions, mediate targeted clearance of CD47‐high apoptotic cells, and release β‐CD to promote cholesterol removal, LXR signaling activation, cholesterol efflux, and inflammatory suppression. Reproduced with permission [70]. Copyright Year 2024. Wiley‐Blackwell.

#### 4.2.3 Skin Scarring and Intervertebral Disc Degeneration

A microneedle‐delivered DPP4 CAR‐TREM2‐M platform locally cleared scar‐associated fibroblasts and reduced hypertrophic‐scar thickness and stiffness [68] (Figure 4D). This preclinical study illustrates how local delivery may confine CAR‐M activity while exploiting macrophage phagocytic and regulatory functions.

#### 4.2.4 Applications of CAR‐M Cell Therapy in Inflammatory Diseases

CAR‐M applications in inflammatory diseases are mainly represented by two strategies: TNF‐responsive switchable CAR‐M and CD47‐targeted CAR‐M.

TNF‐responsive switchable CAR‐M cells localized to inflammatory lesions and improved measures of renal and liver injury in preclinical models [60] (Figure 5B). Their cross‐organ activity supports modular inflammatory sensing, but broad TNF responsiveness also requires careful assessment of host‐defense and stage‐specific effects. In the context of intervertebral disc degeneration, BAI1‐enhanced CAR‐engineered macrophages (CAR‐eMs) were used to augment efferocytosis [69] (Figure 5C), thereby clearing apoptotic nucleus pulposus cells and inflammatory debris in degenerated discs. This approach preserved the viability of residual nucleus pulposus cells and slowed the loss of intervertebral disc height. Together, these two examples highlight a CAR‐M therapeutic paradigm based on “local delivery plus local effector function.”

In atherosclerosis, CD47 CAR‐M cells were combined with HPβ‐CD nanoparticles to couple phagocytic recognition with cholesterol efflux and suppression of foam‐cell conversion [70] (Figure 5D). This dual strategy addresses both defective efferocytosis and lipid burden, but its vascular safety and translational manufacturability remain preclinical questions [71].

CAR‐M translation is constrained by limited primary‐cell expansion, inefficient transduction and complex GMP culture. Clinical experience remains confined to oncology, where early trials such as CT‐0508 provide preliminary safety information; no CAR‐M trial has yet established efficacy in fibrosis. Accordingly, current fibrosis applications should be considered preclinical foundations for, rather than evidence of, clinical readiness.

## 5 CAR‐NK Cell Therapy: A New Option for Safety and Accessibility

Natural killer (NK) cells, as effector‐cell carriers for CAR engineering, possess a series of intrinsic safety attributes that are not shared by T cells or macrophages. These properties arise from the innate immunobiology of NK cells [72]. Their non‐MHC‐restricted cytotoxicity makes NK cells naturally suited for allogeneic applications. NK‐cell activation is regulated by the dual recognition of “missing‐self,” defined by the loss of MHC class I molecules, and “induced‐self,” characterized by the upregulation of stress ligands, rather than by TCR‐mediated recognition of MHC–peptide complexes. Therefore, when donor NK cells are infused into HLA‐mismatched recipients, they generally do not recognize normal host tissues as “non‐self,” thereby mechanistically reducing the molecular basis for graft‐versus‐host disease (GVHD). This feature contrasts sharply with allogeneic CAR‐T cells, which often require CRISPR‐mediated knockout of multiple genes, such as TRAC, B2M, and CIITA, to be safely used in allogeneic settings [73]. Beyond CAR‐dependent cytotoxicity, CAR‐NK cells retain innate killing through CD16, NKG2D and natural cytotoxicity receptors, which may partly compensate for heterogeneous antigen expression [74, 75].Their lower IL‐6 production and lack of TCR‐mediated alloreactivity generally support a favorable acute‐safety profile, although evidence outside oncology remains limited [76].

Limited in vivo persistence is a context‐dependent limitation of CAR‐NK therapy rather than an exclusively unfavorable property. Compared with CAR‐T cells, many CAR‐NK products show weaker expansion and shorter persistence because of limited cytokine support, differentiation state, host immune rejection and reduced metabolic fitness. Early disappearance may permit the recovery of pathogenic B cells, plasma cells or activated fibroblasts, resulting in incomplete disease control or a requirement for repeated administration. Conversely, rapid contraction may reduce prolonged depletion of normal target‐expressing cells, uncontrolled expansion and difficult‐to‐reverse late toxicity, making it potentially advantageous when transient intervention is desired. Persistence also differs substantially among NK‐cell sources: irradiated NK‐92 cells are inherently short‐lived, whereas peripheral‐blood‐, cord‐blood‐ and iPSC‐derived products may display different expansion and survival kinetics. IL‐15 armoring, cytokine‐receptor engineering, memory‐like differentiation and repeat dosing can improve durability, but constitutive cytokine signaling may introduce excessive proliferation or systemic inflammatory toxicity. Thus, CAR‐NK persistence should be matched to disease chronicity, target‐cell regeneration and the required therapeutic window rather than maximized indiscriminately [77].

### 5.1 Sources of NK Cells and Engineering Strategies

#### 5.1.1 Comparison of Five NK Cell Sources

The cellular source of CAR‐NK products directly determines their functional properties, manufacturing feasibility, and clinical positioning. The five major sources currently under investigation each have distinct advantages and limitations (Table 3). Peripheral blood‐derived NK cells retain a mature phenotype and CD16‐mediated antibody‐dependent cellular cytotoxicity, but donor variability and limited expansion restrict standardized production. Cord blood‐ and placenta‐derived NK cells offer greater availability and proliferative potential, although their functional maturity and manufacturing reproducibility require further validation. NK‐92 cells are readily expanded and transduced but lack endogenous CD16, originate from a malignant cell line and require irradiation before infusion, thereby limiting persistence. iPSC‐derived NK cells enable clonally defined and multiplex‐engineered production from master cell banks, but differentiation maturity, genomic stability and manufacturing complexity remain important concerns [78, 79].

**TABLE 3:** Comparison of NK/CAR‐NK Cell Sources and Translational Features.

Source	Advantages	Limitations	Representative applications / translational positioning
Peripheral blood NK (PB‐NK)	Functionally mature, with relatively strong natural cytotoxicity; relatively high CD16 expression and therefore good ADCC capacity; feasible from either autologous or allogeneic sources.	Substantial donor‐to‐donor variability; expansion, transduction efficiency, and post‐cryopreservation viability may be unstable; in vivo persistence is relatively limited.	A conventional autologous or allogeneic CAR‐NK platform; well suited to rapid translation and individualized or donor‐derived studies.
Cord blood NK (CB‐NK)	Relatively standardized source that can be selected from cord blood banks; younger cells with better expansion potential; convenient for allogeneic application.	Baseline phenotype is relatively immature and usually requires ex vivo maturation and activation; ADCC, perforin/granzyme expression, and killing function often need further enhancement.	Widely explored for off‐the‐shelf CAR‐NK products; suitable for combination with IL‐15, iCasp9, safety switches, and related modules.
NK‐92 cell line	Unlimited expansion; amenable to gene editing and clonal selection; high batch consistency; controllable manufacturing cost and process.	Derived from a lymphoma cell line and therefore usually requires irradiation before clinical use; lacks endogenous CD16 and thus has weak ADCC capacity; in vivo persistence is short and repeated infusion may be required.	A common platform in preclinical and early clinical CAR‐NK studies; for example, FAP CAR‐NK‐92 has been used in fibrosis models.
iPSC‐derived NK (iPSC‐NK)	A monoclonal master cell bank can be established, making this source suitable for large‐scale, standardized, off‐the‐shelf production; facilitates multiplex gene editing; product consistency is relatively good.	Differentiation systems are complex and costly; maturity, functional stability, and long‐term safety still require validation; production cycles are relatively long.	An important direction for off‐the‐shelf CAR‐NK development; QN‐139b represents an iPSC‐derived CD19/BCMA dual‐target CAR‐NK explored clinically in systemic sclerosis.
Placental/decidual‐derived NK	NK cells are abundant in placental or decidual tissues and display features related to immunoregulation and tissue remodeling; in theory, they may provide distinctive immune phenotypes.	The research foundation and standardized manufacturing workflows remain insufficient; compared with PB‐NK and CB‐NK, evidence for CAR engineering and clinical translation is limited; functional heterogeneity and source‐related ethical/quality‐control issues remain to be addressed.	Best positioned as a potential or exploratory source rather than a mature CAR‐NK platform for prioritized development.

#### 5.1.2 Design Features of CAR Constructs in CAR‐NK Cells

CAR‐NK constructs share the basic architecture of CAR‐T cells but increasingly incorporate signaling modules adapted to endogenous NK‐cell pathways. In addition to CD3ζ, CD28 and 4‐1BB, NK‐associated domains such as 2B4, DAP10 and DAP12 can enhance activation, degranulation and cytotoxicity through pathways more compatible with intrinsic NK‐cell biology [80]. IL‐15 armoring may improve survival and persistence, although sustained cytokine signaling requires careful control because it may increase abnormal proliferation and inflammatory toxicity.

QN‐139b provides a representative example of multiplex‐engineered iPSC‐derived CAR‐NK cells for immune‐mediated disease [81]. Its design combines B2M and CIITA knockout, HLA‐E/HLA‐G knock‐in, CD16 knockout, IL‐2RF insertion, a tEGFR safety switch and dual CD19/BCMA targeting. These modifications are intended to reduce host immune rejection, support persistence, limit nonspecific activation, broaden pathogenic‐cell coverage and enable emergency elimination [82].

### 5.2 Applications of CAR‐NK Cell Therapy in Autoimmune Diseases

QN‐139b represents an early proof‐of‐concept for the application of CAR‐NK therapy in autoimmune diseases [81] (Figure 6A). This multiplex‐engineered, CD19/BCMA‐targeted iPSC‐derived CAR‐NK product was administered to one patient with severe diffuse cutaneous systemic sclerosis. Treatment induced profound B‐cell depletion in peripheral blood and bone marrow, accompanied by improvement in the modified Rodnan skin score and partial improvement in microvascular parameters. Only mild cytokine release syndrome was reported, without ICANS or graft‐versus‐host disease. Single‐cell transcriptomic and proteomic analyses suggested remodeling of immune‐inflammatory and fibrosis‐associated pathways. However, a single‐patient observation cannot establish general efficacy, durability, relapse risk or the frequency of rare adverse events.

**FIGURE 6:**
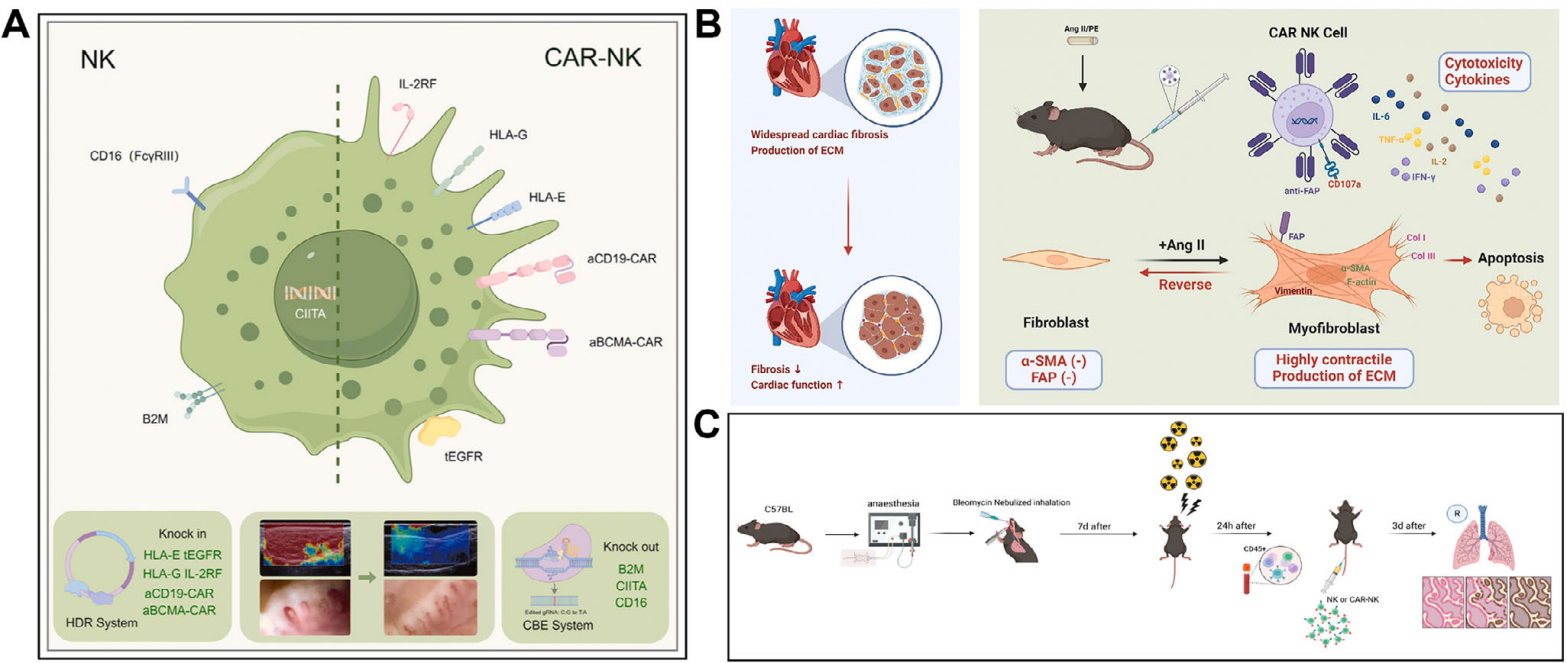
Schematic illustration of CAR‐NK therapy in fibrotic diseases. (A) Schematic illustration of QN‐139b, an iPSC‐derived CD19/BCMA dual‐targeting CAR‐NK product, for severe refractory systemic sclerosis. QN‐139b showed minimal toxicity, depleted pathogenic B cells, reduced autoantibodies, induced immune resetting, and promoted the recovery of fibrotic skin and vascular structures. Reproduced with permission [81]. Copyright Year 2025. Cell Press. (B) Schematic illustration of FAP‐targeted CAR‐NK cells for alleviating cardiac fibrosis. FAP CAR‐NK cells recognize FAP‐positive myofibroblasts, secrete effector cytokines, reverse profibrotic and hypercontractile myofibroblast phenotypes, and induce apoptosis of target cells, thereby reducing fibrotic area and improving cardiac function in vitro and in vivo. Reproduced with permission [83]. Copyright Year 2025. Elsevier. (C) Schematic of the experimental design, including bleomycin administration, fractionated total body irradiation (TBI), infusion of PBS, untransduced NK cells, or FAP‐CAR‐NK cells with rhIL‐15 support, and acute tissue collection. Reproduced with permission [84]. Copyright Year 2026. MDPI (Basel, Switzerland).

### 5.3 Applications of CAR‐NK Cell Therapy in Fibrotic Diseases

In a murine cardiac‐fibrosis model, irradiated FAP CAR‐NK‐92 cells reduced activated fibroblasts, collagen deposition and fibrotic area with only mild acute toxicity [83] (Figure 6B). However, irradiation prevents proliferation and sharply limits persistence, making durability and repeat dosing central translational constraints. More recently, Wu et al. further extended the FAP‐targeted CAR‐NK strategy from the NK‐92 cell line to PBMC‐derived primary human NK cells and performed proof‐of‐concept validation in pulmonary fibrosis‐related models [84] (Figure 6C). Short‐term trafficking to injured mouse lung provided additional proof of concept, but small samples, irradiation preconditioning and an acute observation window preclude conclusions about durable anti‐fibrotic efficacy or systemic safety.

Taken together, CAR‐NK is best viewed as a distinct platform for short‐term, controllable clearance: limited persistence may reduce prolonged toxicity but may also compromise durability and require repeat dosing [85].

## 6 Comparison of the Three Major CAR Platforms and Progress in Clinical Translation

### 6.1 Effector Mechanisms

As summarized in Table 4, the three major CAR platforms harness distinct immune effector mechanisms to eliminate pathogenic targets and modulate disease‐associated microenvironments, and these mechanistic differences directly shape their respective therapeutic applications (Figure 7).

**TABLE 4:** Comparison of Effector Mechanisms Across Three CAR Platforms.

Dimension	CAR‐T	CAR‐M	CAR‐NK
Core effector mechanism	Antigen‐dependent cytotoxic killing, mainly mediated through perforin/granzyme release, Fas/FasL signaling, and inflammatory cytokine‐driven target‐cell clearance.	Antigen‐dependent phagocytic clearance, efferocytosis/corpse‐clearance‐like activity, antigen processing, and macrophage polarization reprogramming.	CAR‐dependent killing plus cooperation with natural NK receptors, such as NKG2D and NCRs; CD16‐positive cells may additionally mediate ADCC.
ECM degradation / matrix remodeling capacity	Usually lacks direct ECM‐degrading capacity, and primarily alters the microenvironment indirectly through elimination of pathological cells.	Relatively strong matrix‐remodeling potential; macrophages can produce MMPs, and CD147‐based CAR designs can further enhance MMP‐mediated ECM degradation.	Usually lacks direct ECM‐degrading capacity, and primarily influences the microenvironment indirectly through killing of pathological cells and cytokine secretion.
Antigen presentation capacity	Not a professional antigen‐presenting cell; CAR recognition itself is MHC‐independent, but CAR‐T does not serve as a major APC platform.	Relatively strong; can activate CD4+ T cells through MHC II, co‐stimulatory molecules, and antigen processing, and may also participate in cross‐presentation.	Usually not considered a professional APC, but may promote DC‐ and T‐cell responses through factors such as IFN‐γ.
Immune memory / long‐term surveillance	Relatively strong; can generate central memory or stem‐like memory T cells and thereby support long‐term immune surveillance.	Lacks classical adaptive immune memory, but may exhibit tissue residency and trained‐immunity‐like reprogramming features.	Generally weaker than CAR‐T; some NK or CAR‐NK products may display adaptive‐ or memory‐like features, but their stability and durability remain limited.
In vivo persistence	Relatively strong, ranging from months to years; persistence associated with 4‐1BB is generally superior to that associated with CD28.	Usually short to intermediate, and influenced by cellular source, polarization state, tissue engraftment, and the local microenvironment.	Highly source‐ and engineering‐dependent; generally less predictable than CAR‐T, whereas IL‐15 armoring can markedly enhance persistence.
Cytokine secretion profile	Primarily includes IFN‐γ, TNF‐α, IL‐2, and GM‐CSF; IL‐6 and IL‐1 observed in CRS are often amplified mainly by the monocyte‐macrophage system rather than being directly produced at high levels by CAR‐T cells themselves.	Polarization‐dependent: M1‐like states may secrete IL‐12, TNF‐α, IL‐1β, IL‐6, and related mediators, whereas M2/reparative states may produce IL‐10, TGF‐β, and related factors.	Primarily includes IFN‐γ, TNF‐α, and GM‐CSF; the overall CRS risk is usually lower than that of CAR‐T, and IL‐6 is not generally regarded as a defining signature cytokine.
Tissue infiltration / residency	Infiltration into solid or fibrotic tissues is relatively constrained, and often requires chemokine‐receptor engineering or local delivery strategies for enhancement.	Relatively strong; macrophages are naturally inclined toward tissue infiltration, lesion recruitment, and tissue residence, making them well suited to inflammatory/fibrotic microenvironment remodeling.	Intermediate; influenced by NK source, chemokine‐receptor expression, and the local immunosuppressive milieu.
Major safety concerns	CRS, ICANS, on‐target/off‐tumor toxicity, prolonged B‐cell aplasia, and related complications.	Non‐specific phagocytosis, excessive inflammatory activation, M2‐like profibrotic deviation, and tissue‐injury risk.	On target/off‐tissue cytotoxicity; CRS and neurotoxicity are generally less frequent, whereas variable persistence, manufacturing consistency, and possible repeat dosing remain translational challenges.

**FIGURE 7:**
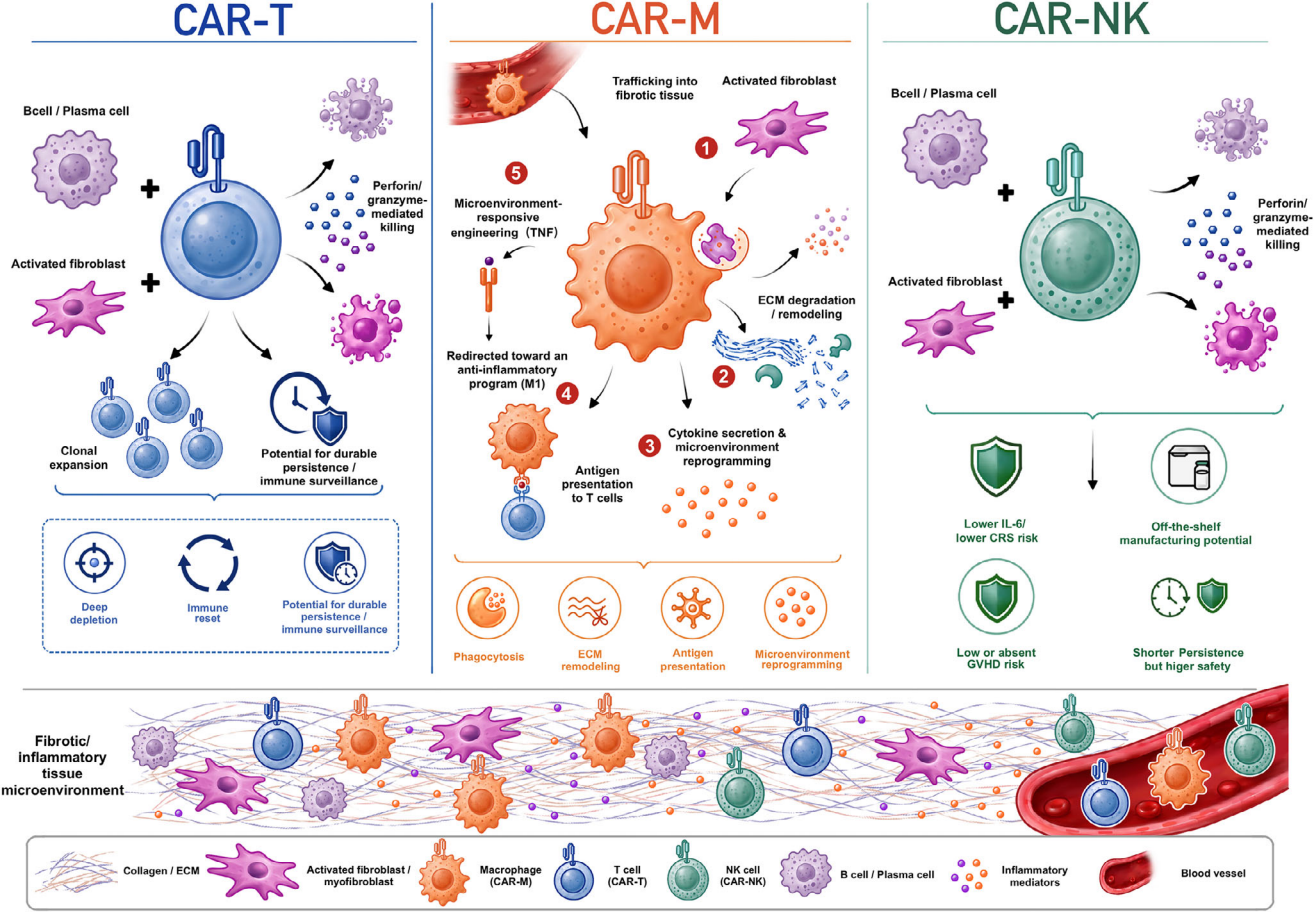
Schematic illustration of the effector mechanisms of the three major CAR platforms: CAR‐T, CAR‐M, and CAR‐NK cells.

CAR‐T cells mainly exert their effector function through antigen‐dependent, contact‐mediated cytotoxicity. After antigen recognition, CAR clustering triggers CD3ζ ITAM phosphorylation and downstream T‐cell activation signaling, leading to immunological synapse formation, cytotoxic granule polarization, and release of perforin and granzymes to induce target‐cell apoptosis. CAR‐T cells may also eliminate target cells through death receptor pathways, including Fas/FasL and TRAIL, and can sequentially kill multiple target cells through serial killing. Beyond direct cytotoxicity, activated CAR‐T cells secrete cytokines such as IFN‐γ, TNF‐α, and GM‐CSF, which amplify local immune responses and modulate myeloid, stromal, and bystander immune cells. Their capacity to generate memory‐like populations and persist long term further enables sustained immune surveillance after antigen re‐emergence. In non‐oncological diseases, however, these same properties—potent cytotoxicity, inflammatory amplification, and durable persistence—represent both therapeutic advantages and potential safety risks. Therefore, CAR‐T activity should be carefully modulated through target selection, affinity tuning, transient expression, logic gating, or controllable safety switches [86, 87].

CAR‐M cells exert their major effects through targeted phagocytosis and microenvironmental remodeling rather than classical cytotoxic killing. Upon antigen recognition, intracellular modules such as FcRγ, CD3ζ, or Megf10 can activate macrophage phagocytic programs, enabling the clearance of pathological cells, apoptotic debris, and damage‐associated components. In fibrotic or chronically inflamed lesions, CAR‐M cells may further influence fibroblasts, endothelial cells, and endogenous immune populations by secreting immunoregulatory cytokines, chemokines, and matrix‐remodeling mediators. Their therapeutic value therefore extends beyond eliminating activated fibroblasts or other pathogenic cell populations to include regulation of ECM deposition, tissue remodeling, and local inflammatory tone. In addition, macrophages possess intrinsic antigen‐processing and MHC class II antigen‐presentation capacity, and CAR‐directed phagocytosis may enhance antigen presentation and secondary immune activation. Compared with CAR‐T cells, CAR‐M cells generally lack classical immune memory, show more limited persistence, and remain highly responsive to tissue‐derived cues. Thus, they are particularly suited for localized diseases requiring coordinated targeted clearance, inflammatory regulation, and matrix remodeling, such as fibrosis, chronic inflammation, and dysregulated tissue repair. Their long‐term efficacy, however, may require repeated dosing, local delivery, survival‐enhancing engineering, or combination with strategies that promote endogenous immune remodeling [88].

CAR‐NK cells combine CAR‐dependent antigen recognition with the innate cytotoxic programs of natural killer cells. After target engagement, they can induce apoptosis through perforin/granzyme release and cytokine secretion, while their signaling design may incorporate NK‐adapted modules such as DAP10, DAP12, 2B4, or NKG2D in addition to conventional CD3ζ‐, CD28‐, or 4‐1BB‐based structures. Importantly, CAR‐NK cells retain CAR‐independent recognition pathways, including activating receptors such as NKG2D, DNAM‐1, natural cytotoxicity receptors, and CD16‐mediated ADCC, which may provide complementary killing capacity when target‐antigen expression is heterogeneous or partially lost. Compared with CAR‐T cells, CAR‐NK cells are generally associated with lower risks of GVHD and severe cytokine release syndrome and are well suited to allogeneic, off‐the‐shelf manufacturing. However, their persistence is usually shorter, and NK cells from different sources vary in expansion potential, maturation state, CD16 expression, and in vivo survival. Thus, CAR‐NK cells may be particularly useful when short‐term, controllable pathological cell clearance is preferred over durable immune surveillance, especially in inflammatory or fibrotic diseases where safety and accessibility are prioritized [89].

Therefore, CAR‐T, CAR‐M, and CAR‐NK cells should not be viewed as simple substitutes for one another. Rather, they represent three distinct engineered cell‐therapy logics: “sustained immune surveillance,” “microenvironmental remodeling,” and “short‐term safe clearance,” respectively. For non‐oncological diseases, the therapeutic goal is often not to maximize cytotoxicity, but to establish a balance among pathological cell clearance, inflammatory control, tissue repair, and long‐term safety.

### 6.2 Acute, Long‐Term and Platform‐Specific Safety

Safety is one of the central determinants of whether CAR therapy can be broadly implemented in non‐oncological diseases (Table 5). The safety risks of CAR‐T therapy appear to be substantially lower in non‐oncological patients than in cancer patients, but they still require careful management. In autoimmune diseases, cytokine release syndrome (CRS) is usually grade 1–2, with grade ≥3 CRS occurring in fewer than 5% of cases, while immune effector cell‐associated neurotoxicity syndrome (ICANS) is rare, occurring in fewer than 5% of patients. During prolonged B‐cell aplasia, typically within 3–6 months after treatment, immunoglobulin replacement may be required. In contrast, tumor‐directed CAR‐T therapy is associated with grade ≥3 CRS rates of 10%–30%, ICANS rates of 10%–20%, and treatment‐related mortality of 1%–4%. The management of ICANS and immune effector cell‐associated hematotoxicity (ICAHT) is generally less challenging in autoimmune patients, who are often younger, have a lower target‐cell burden, and experience less intense CAR‐T activation [90, 91].

**TABLE 5:** Comparison of Safety Considerations Across CAR‐Engineered Immune‐Cell Platforms in Fibrotic and Immune‐Mediated Inflammatory Diseases.

CAR‐engineered cell platform	Acute safety considerations	Long‐term or platform‐specific risks	Allogeneic, immunogenic and genomic risks	Evidence level in the reviewed indications
CAR‐T	In published autoimmune‐disease reports, CRS has been predominantly grade 1‐2, whereas grade ≥3 CRS and ICANS have been uncommon.	For B‐cell‐directed CAR‐T, B‐cell aplasia, hypogammaglobulinemia, infection and ICAHT; durable expansion may prolong on‐target/off‐tissue injury.	Autologous products do not cause GVHD. Allogeneic products require control of TCR‐mediated alloreactivity; receptor immunogenicity and vector‐ or editing‐related genotoxicity remain concerns.	Case reports or series and early‐phase trials in autoimmune diseases; antifibrotic applications remain preclinical; no approved non‐oncological indication.
CAR‐M	Clinical safety data outside oncology are unavailable. Early oncology experience suggests mainly low‐grade CRS and no clear ICANS signal, but patient exposure remains limited.	Phenotypic plasticity may permit maladaptive inflammatory or profibrotic7 repolarization; limited persistence may necessitate repeat dosing.	Classical TCR‐mediated GVHD is not expected, although immune responses to the receptor or vector and manufacturing‐related variability remain possible.	Preclinical evidence in fibrotic and immune‐mediated inflammatory diseases.
CAR‐NK	Early autoimmune‐disease studies suggest a favorable acute safety profile, with mostly absent or low‐grade CRS and no consistent ICANS or GVHD signal reported to date.	Limited expansion and persistence may compromise durability and require repeat dosing; cytokine armoring may introduce proliferative or inflammatory toxicity.	Classical GVHD risk is low, but host rejection, anti‐product immunity and repeat‐dose immunogenicity remain relevant.	Early clinical evidence in autoimmune diseases, including SLE case series/early‐phase studies and SSc proof‐of‐concept; antifibrotic applications remain preclinical.
Unconventional CAR T‐lineage cells (NKT, MAIT and γδ T)	Acute toxicity is not defined in non‐oncological disease; endogenous receptor and cytokine activity could amplify local inflammation.	Subset heterogeneity, variable expansion, uncertain persistence and tissue‐specific trafficking may affect both efficacy and safety.	Reduced dependence on conventional peptide‐MHC recognition may lower GVHD risk, but this is subset specific; endogenous receptors may broaden off‐target activation.	Predominantly preclinical outside oncology.

Based on currently available clinical data, including the phase I trial of CT‐0508, the acute toxicity profile of CAR‐M appears relatively mild. CRS is predominantly low grade, with no reported grade ≥3 events, and ICANS has not been observed. The distinctive concern for CAR‐M lies in the risk of M2‐like repolarization [92]. In fibrotic or TGF‐β‐rich microenvironments, CAR‐M cells may shift toward an immunosuppressive phenotype, potentially leading to loss of anti‐fibrotic activity and, theoretically, promotion of matrix deposition, although this has not yet been demonstrated clinically. Among the three platforms, CAR‐NK cells show the clearest safety advantage, with grade ≥3 CRS rates below 7% and almost no reported ICANS or GVHD, even in HLA‐mismatched allogeneic settings. The intrinsically low IL‐6 secretion and absence of TCR expression in NK cells provide the molecular basis for their favorable safety profile [72, 93].

Beyond CRS, ICANS and GVHD, CAR therapies raise longer‐term concerns involving immunogenicity, insertional or editing‐related genotoxicity, on‐target/off‐tissue injury, prolonged immune suppression and manufacturing variability. These risks warrant particular caution in non‐malignant disease, where tolerance for irreversible toxicity is lower. Immune responses against non‐human binding domains, junctional sequences, vectors or editing proteins may shorten persistence or compromise repeat dosing, even when humanized constructs are used [94].

Integrating viral vectors and transposons provide durable expression but retain risks of insertional mutagenesis and clonal expansion, whereas genome editing can introduce off‐target mutations, large deletions or chromosomal rearrangements, particularly in multiplex‐edited universal products. Long‐term surveillance, integration‐site or genomic analysis and construct‐specific monitoring therefore remain necessary. Target toxicity is equally important because CD19, BCMA and fibrosis‐associated antigens are not restricted to pathogenic cells; sustained targeting may cause prolonged humoral immunodeficiency or disrupt normal stromal repair. Logic gating, affinity tuning, transient expression and safety switches can reduce, but not eliminate, these risks [95].

In vivo systems introduce additional concerns: repeated LNP or viral dosing may provoke anti‐carrier immunity, complement activation, altered biodistribution or accelerated clearance, whereas persistent vectors provide less control after unintended transduction. Because the cellular product is generated inside the patient, conventional pre‐infusion release testing must be replaced by stringent assessment of vector identity, potency, target‐cell selectivity, biodistribution and expression kinetics [96].

Shared risks across CAR‐T, CAR‐NK, CAR‐M and unconventional CAR platforms include receptor immunogenicity, off‐tissue activity, vector‐related genotoxicity, contamination and uncertain persistence, but their consequences are platform specific. CAR‐T expansion and memory can prolong toxicity; CAR‐NK cells generally show lower GVHD risk but limited persistence, which cytokine‐support modules may alter; CAR‐M plasticity can redirect inflammatory or fibrotic activity; and unconventional cells retain endogenous receptor‐mediated activation.

### 6.3 Manufacturing and Accessibility

Manufacturing models are a key determinant of whether CAR therapy can move from “elite medicine” toward routine clinical care (Table 6).

**TABLE 6:** Comparison of ex vivo Manufacturing Models, Quality‐Control Considerations and Accessibility Across CAR Cell Platforms.

Manufacturing model	Workflow and treatment readiness	Scalability and batch consistency	Key CMC characterization and release considerations	Translational relevance and accessibility in the reviewed indications
Autologous CAR‐T	Patient‐specific collection, activation, CAR transduction, expansion and release testing; treatment preparation typically requires several weeks.	Low scalability and relatively high donor‐ and disease‐dependent variability.	Identity, purity, viability, CAR expression, potency, sterility/mycoplasma, vector copy number and replication‐competent virus testing (as applicable), and product stability.	The most clinically mature route in autoimmune disease, but long lead time, high cost and dependence on specialized centers restrict accessibility; direct fibrosis‐targeted use remains preclinical.
Gene‐edited allogeneic CAR‐T	Donor‐cell banking, multiplex editing, expansion and cryopreservation enable a premanufactured product, but increase process and comparability complexity.	Potentially high scalability, although donor‐bank qualification, editing complexity and process comparability affect batch consistency.	Identity, purity, viability, CAR expression, potency and sterility, together with residual TCR/alloreactive cells, editing efficiency, off‐target and chromosomal alterations, genomic integrity and post‐thaw recovery.	Early clinical evidence has emerged in refractory SLE; this model may shorten treatment initiation, but host rejection, editing‐related safety and long‐term surveillance remain unresolved.
Primary CAR‐M	Monocyte isolation, controlled differentiation and engineering are performed ex vivo; limited expansion and engineering efficiency constrain scale and process consistency.	Low to moderate scalability; limited expansion, differentiation efficiency and phenotype stability may contribute to batch variability.	Identity, purity, viability, CAR expression, polarization stability, phagocytic potency, cytokine profile, sterility and product stability.	Non‐oncological use remains preclinical; limited expansion, process complexity and incomplete GMP standardization currently restrict scalability and accessibility.
Donor‐derived CAR‐NK	Peripheral‐blood or cord‐blood NK cells require donor qualification, expansion, engineering and cryopreservation before use.	Moderate to high off‐the‐shelf potential, but source‐dependent yield, maturation, engineering efficiency and expansion can reduce consistency.	Lineage identity, viability, purity, CAR expression, NK‐receptor phenotype, CAR‐dependent and CAR‐independent cytotoxic potency, sterility and post‐thaw function.	Allogeneic production may improve accessibility. Early phase I evidence is now available in refractory SLE using cord blood‐derived CD19 CAR‐NK cells, but durability and generalizability remain uncertain.
iPSC‐derived CAR immune cells (CAR‐NK clinical proof‐of‐concept; CAR‐M preclinical)	Clonal engineering of a master cell bank is followed by controlled differentiation, purification and cryopreservation.	Potentially high scalability and clonal consistency, but differentiation efficiency, terminal maturation and run‐to‐run reproducibility require control.	Master‐cell‐bank identity and qualification, genomic and clonal stability, residual pluripotency, lineage maturation, purity, potency, sterility and post‐thaw recovery.	May provide a highly scalable and standardized off‐the‐shelf route. A single‐patient report in systemic sclerosis supports early feasibility for iPSC‐CAR‐NK, whereas iPSC‐CAR‐M remains preclinical; durable activity, long‐term safety and realized cost reduction remain unproven.

Autologous CAR‐T manufacturing is currently the most mature model, but it is also among the most expensive and operationally complex. Cell production typically requires 2–6 weeks, and the listed prices of commercially approved CAR‐T products in the United States generally fall within the range of approximately US$370 000–500 000 per dose, excluding additional medical costs associated with hospitalization, toxicity management, and long‐term follow‐up [97]. For patients with end‐stage fibrosis, this waiting period may translate into further, and potentially irreversible, loss of organ function. Allogeneic CAR‐T strategies are being developed to reduce TCR‐mediated alloreactivity and host immune rejection through multiplex CRISPR editing. For example, the multi‐gene editing strategy represented by TyU19 has shown early feasibility in severe autoimmune diseases; however, its manufacturing complexity is higher, and risks related to gene‐editing off‐target effects, chromosomal structural abnormalities, and long‐term safety require rigorous evaluation [14, 43, 98].

CAR‐M faces manufacturing challenges distinct from those of CAR‐T. Primary macrophages have limited ex vivo expansion capacity, relatively low viral transduction efficiency, and more complex GMP culture requirements than T cells. iPSC‐derived CAR‐M or CAR‐iMAC may provide a route toward scalable and standardized production, but the consistency of differentiation efficiency, terminal maturation, tissue adaptability, and long‐term safety still require optimization. By contrast, CAR‐NK has intrinsic advantages for allogeneic off‐the‐shelf manufacturing, especially through iPSC‐derived routes, which can rely on clonal master cell banks to enable large‐scale, homogeneous, and multiplex gene‐edited production. This approach may reduce donor variability and batch‐to‐batch heterogeneity while improving therapeutic accessibility. However, its actual production cost, in vivo persistence, differentiation maturity, genomic stability, and long‐term safety still require further validation through clinical‐grade manufacturing and multicenter studies. Therefore, CAR‐NK occupies a distinctive position in the narrative of “democratizing cell therapy;” at present, however, it is more appropriate to describe it as a platform with cost‐reduction potential and scalability prospects, rather than to prematurely assign a specific expected cost per dose [99, 100]. Release testing should therefore combine common measures of identity, purity, viability, CAR expression, potency, sterility and genomic integrity with platform‐specific assays of T‐cell differentiation, NK‐cell receptor and post‐thaw function, macrophage polarization and phagocytosis, and residual pluripotency in iPSC‐derived products.

### 6.4 In Vivo CAR Engineering: Delivery Technologies and Delivery‐Related Translational Challenges

In vivo CAR engineering generates CAR‐expressing immune cells directly within the patient by delivering CAR‐encoding nucleic acids or genome‐editing components to endogenous cell populations. By avoiding leukapheresis and individualized ex vivo activation, transduction and expansion, this approach may shorten treatment initiation and improve scalability. However, it also shifts control of product identity, dose and phenotype from a defined cellular product to an in situ process governed by vector biodistribution, target‐cell abundance, transduction efficiency and expression kinetics. In vivo engineering should therefore be considered a distinct therapeutic modality rather than simply a manufacturing shortcut [96].

Targeted mRNA lipid nanoparticles currently represent the most developed non‐viral strategy. Ligands directed against T‐cell markers can enrich delivery to selected subsets, whereas the non‐integrating and transient nature of mRNA enables reversible CAR expression. CD5‐targeted LNPs generated transient FAP‐directed CAR‐T cells in mice, reduced cardiac fibrosis and improved function. More recently, targeted LNPs reprogrammed CD8+ T cells from healthy donors and patients with autoimmune disease and induced B‐cell depletion in cynomolgus monkeys [101]. Nevertheless, transient expression may require repeated administration, with potential risks of innate immune activation, anti‐carrier antibodies, complement activation, altered biodistribution and accelerated clearance.

Retargeted viral vectors offer an alternative when efficient and durable expression is required. Their potential for sustained CAR generation must be balanced against insertional mutagenesis, unintended transduction, vector shedding and limited reversibility. Virus‐like particles, enveloped delivery vehicles and polymeric or fusogenic nanoparticles broaden cargo and tissue‐targeting options but introduce distinct constraints involving payload capacity, pre‐existing immunity, manufacturing consistency and repeat dosing [102].

Delivery specificity may be reinforced by immune‐cell‐specific promoters, microRNA‐mediated detargeting or other post‐entry controls. Gene‐editing systems may enable site‐specific CAR insertion and more uniform expression, but off‐target mutations, large deletions and chromosomal rearrangements remain barriers. Translation will therefore require quantitative control of biodistribution, target‐cell selectivity, expression duration and the phenotype of CAR‐expressing cells generated in vivo. Because no conventional cellular product is available for pre‐infusion release testing, quality control must emphasize vector identity, purity and potency, non‐target transduction and long‐term genomic and immunological surveillance.

### 6.5 Decision‐Making Framework for Context‐Specific Application

Based on the above comparison, we propose a scenario‐matching decision logic built on four key factors.

First, the pathogenic mechanism should guide platform selection. Systemic autoimmune diseases driven by B cells or plasma cells, such as SLE, SSc, RA, MS, and myositis, are naturally more suitable for CAR‐T therapy, as deep and durable B‐cell depletion can induce immune resetting. In contrast, local fibrotic diseases, including cardiac, hepatic, pulmonary, and renal fibrosis, are more naturally aligned with CAR‐M therapy, given its irreplaceable capacity for multidimensional microenvironmental remodeling [35].

Second, the tissue microenvironment must be considered. Lesions characterized by dense ECM, ischemia, hypoxia, and abundant immunosuppressive factors may require the ECM‐penetrating and anti‐repolarization capacities of CAR‐M cells. By contrast, systemically distributed pathogenic cells, such as B cells residing across the bone marrow, lymph nodes, and peripheral blood, are better suited to the systemic surveillance and deep clearance capacity of CAR‐T cells [103].

Third, safety requirements should influence the therapeutic choice. For elderly, frail, or highly comorbid patients, or for those with shorter life expectancy or unwillingness to accept infection risks associated with B‐cell aplasia, CAR‐NK may represent the safest option. For younger patients with refractory autoimmune disease who have failed conventional therapies and urgently require deep remission, the benefit–risk profile of CAR‐T therapy may be acceptable.

Fourth, accessibility constraints are critical. In regions with limited resources or without access to CAR‐T manufacturing centers, LNP‐mediated in vivo generation of CAR‐T or CAR‐M cells, or iPSC‐derived off‐the‐shelf CAR‐NK products, may represent more realistic options.

Collectively, platform selection should be context specific, matching the pathogenic compartment, tissue accessibility, required persistence, safety tolerance and manufacturing feasibility rather than following a single hierarchy.

### 6.6 Current Clinical Evidence and Translational Status

To avoid conflating therapeutic promise with clinical maturity, the evidence discussed here is classified as approved, under clinical investigation, reported in individual cases or case series, or supported only by preclinical studies. No CAR therapy is currently approved for a non‐oncological indication. In autoimmune diseases, published clinical evidence consists mainly of case reports and small case series, while an increasing number of early‐phase trials are evaluating CAR‐based approaches across SLE, SSc, RA, MS, myositis, myasthenia gravis and related disorders. CD19 remains the dominant target, whereas BCMA‐targeted and CD19/BCMA or CD19/CD20 dual‐target strategies are less clinically mature. Representative investigational products include KYV‐101, CABA‐201, YTB323 and TyU19. The reported use of the CD19/BCMA‐targeted iPSC‐derived CAR‐NK product QN‐139b in a single patient with SSc should be interpreted as proof‐of‐concept rather than established clinical efficacy [104].

For fibrosis, the evidence remains preclinical, and no registered interventional trial of a CAR cell product was identified as of the literature‐search cut‐off date. FAP‐, uPAR‐ and PDGFRβ‐directed approaches have shown activity in animal models of cardiac, hepatic, pulmonary or renal fibrosis, but differences in antigen distribution, disease stage, model duration and safety assessment limit direct comparison. LNP‐mRNA‐mediated in vivo CAR generation and local delivery should therefore be described as preclinical enabling technologies rather than clinical‐stage therapies; their technical development is discussed in Section 6.4. Proposed first‐in‐human evaluation in advanced fibrosis remains a prospective strategy that will require human target validation, biodistribution studies and predefined stopping rules rather than evidence of current clinical readiness [105].

### 6.7 Other CAR‐Engineered Immune Cells: Expanding the Scope

Beyond CAR‐T, CAR‐M, and CAR‐NK cells, CAR engineering is being extended to unconventional T cells, regulatory and innate immune cells, and stem‐cell‐derived cellular products. Rather than simply reproducing the cytotoxic paradigm of conventional CAR‐T cells, these emerging platforms aim to match distinct cellular functions—including immune tolerance, tissue homing and transient inflammatory recruitment—to specific disease mechanisms [106, 107].

#### 6.7.1 Unconventional T‐Cell Platforms: CAR‐NKT, CAR‐MAIT and CAR‐γδ T Cells

Conventional αβ T cells remain the predominant cellular substrate for CAR engineering; however, unconventional T‐cell populations, including natural killer T (NKT), mucosal‐associated invariant T (MAIT), and γδ T cells, are increasingly being explored as alternative effector platforms. These cells combine CAR‐dependent antigen recognition with endogenous immune functions mediated through non‐classical antigen‐presentation systems or largely MHC‐independent recognition pathways. Selected subsets exhibit restricted alloreactivity, tissue tropism, and innate‐like responses that may support allogeneic product development and provide advantages in diseases characterized by tissue‐localized inflammation or fibrosis. However, these properties differ substantially among subsets and disease contexts and should not be interpreted as conferring uniformly superior efficacy or safety.

NKT cells recognize glycolipid antigens presented by the non‐polymorphic CD1d molecule and can rapidly produce cytokines, recruit other immune cells, and interact with myeloid and stromal compartments. Invariant NKT cells are particularly attractive for allogeneic CAR therapy because their restricted T‐cell receptor repertoire is associated with a low risk of graft‐versus‐host disease. CAR‐NKT cells can integrate CAR‐mediated targeting with endogenous NKT‐cell receptor and NK‐receptor activity, providing complementary modes of target recognition and potential modulation of the surrounding immune microenvironment. A clinically oriented culture system has enabled high‐yield generation of allogeneic CAR‐NKT cells from hematopoietic stem and progenitor cells, with preclinical evidence of expansion, persistence, reduced immunogenicity, and no detectable graft‐versus‐host disease [108]. More recently, allogeneic CAR‐NKT cells were shown to combine CAR‐, TCR‐, and NK‐receptor‐mediated activity with tissue homing and microenvironmental modulation in ovarian cancer models. Although these studies were conducted primarily in oncology, their demonstrated interactions with myeloid and stromal compartments may also be relevant to immune‐mediated and fibrotic diseases.

MAIT cells recognize riboflavin‐pathway‐derived microbial metabolites presented by the highly conserved MR1 molecule and are enriched in the liver, lung, intestine, and other mucosal tissues. Their tissue distribution, rapid cytokine responses, and limited dependence on polymorphic MHC molecules provide a rationale for developing allogeneic CAR‐MAIT products. CAR expression could add disease‐antigen specificity while preserving MR1‐dependent recognition and cytokine‐driven activation. This combination may be advantageous in diseases in which pathogenic immune or stromal cells reside within mucosal or metabolically active tissues. However, MAIT cells can adopt pro‐inflammatory, cytotoxic, regulatory, or tissue‐repair‐associated phenotypes depending on the cytokine milieu and disease stage. Their low frequency in peripheral blood, donor‐dependent expansion, functional heterogeneity, and susceptibility to chronic activation remain substantial manufacturing and biological challenges.

γδ T cells recognize phosphoantigens, stress‐associated ligands, and other non‐peptide targets largely independently of conventional peptide–MHC presentation. Circulating Vγ9Vδ2 T cells and tissue‐enriched Vδ1 T cells differ in antigen recognition, tissue trafficking, expansion, and persistence, creating opportunities for disease‐ and organ‐specific product design. CAR‐γδ T cells may retain endogenous recognition of stressed or activated cells while adding CAR‐dependent specificity, potentially complementing CAR recognition when disease‐associated antigen expression is heterogeneous or dynamic. Their relatively low propensity to induce graft‐versus‐host disease also supports allogeneic development. Conversely, subset heterogeneity, inconsistent expansion, sensitivity to prolonged stimulation, and uncertainty regarding persistence complicate the establishment of standardized products [109].

These unconventional T‐cell platforms should not be viewed as a single interchangeable category. CAR‐NKT cells may be most attractive when allogeneic use and microenvironmental modulation are priorities; CAR‐MAIT cells may be suited to diseases involving liver, lung, intestinal, or other mucosal tissues; and CAR‐γδ T cells may be advantageous when stress‐associated recognition and tissue‐resident immunity are relevant. However, direct evidence in autoimmune and fibrotic diseases remains limited, and endogenous receptor activity may either complement CAR function or amplify local inflammation. Future development will require subset‐specific manufacturing, potency assays that capture both CAR‐dependent and endogenous functions, control of persistence and cytokine production, and validation in chronic disease models that reproduce human tissue microenvironments.

#### 6.7.2 Regulatory and Innate CAR‐Engineered Platforms: CAR‐Treg, iCDC and CAR‐Neutrophils

CAR engineering is also being extended to regulatory immune cells whose primary function is immune‐tolerance restoration rather than target‐cell elimination. CAR‐Tregs use FOXP3^+^ regulatory T cells to deliver antigen‐directed suppressive activity to defined tissues or immune environments, supporting their potential application in autoimmune disease, transplant rejection and type 1 diabetes. Their translation nevertheless depends on stable lineage identity, preserved suppressive function and selective tissue targeting. Engineered immunosuppressive dendritic cells provide a related local‐reprogramming strategy. FAP‐targeted iCDCs expressing CTLA4‐Ig, PD‐L1 and IL‐10 can accumulate in fibrotic cardiac lesions, suppress excessive innate and stromal‐cell activation and promote local Treg expansion. Although anti‐inflammatory and anti‐fibrotic effects have been demonstrated in preclinical models, their durability, biodistribution and potential for excessive immunosuppression require further evaluation.

By contrast, CAR‐neutrophils are designed to exploit the rapid recruitment of neutrophils to inflamed tissues and may therefore support short‐duration intervention in acute inflammation, infection or tissue injury. However, their short lifespan, limited ex vivo expansion and unstable engineering remain major manufacturing constraints, leaving this platform largely at the proof‐of‐concept stage. Together, these approaches extend CAR engineering toward antigen‐directed immune tolerance, local microenvironmental reprogramming and transient inflammatory homing.

#### 6.7.3 iPSC‐Derived CAR Immune Cells as Scalable Off‐the‐Shelf Platforms

iPSCs offer a renewable, clonally defined source for standardized CAR immune‐cell products. CAR‐NK is currently the most advanced lineage, whereas iPSC‐derived CAR‐M, CAR‐NKT, CAR‐γδ T and CAR‐MAIT products remain preclinical and face lineage‐specific maturation and consistency challenges. Compared with donor‐derived products, iPSC‐derived cells offer renewable starting material, clonal consistency and greater flexibility for multiplex engineering before lineage differentiation. Conversely, donor‐derived cells are generally more mature, require fewer differentiation steps and avoid the risk of residual pluripotency. Clinical‐grade iPSC manufacturing therefore requires master‐cell‐bank characterization, genomic and clonal screening, controlled differentiation, purification and cryopreservation. Product release should assess genomic stability, identity, purity, maturation, residual pluripotent cells, CAR expression, potency and post‐thaw recovery. Whether these manufacturing advantages ultimately reduce total cost or improve clinical outcomes remains uncertain [110].

Compared with donor‐derived products, iPSC‐derived cells provide renewable starting material, greater clonal control, and increased flexibility for multiplex engineering before lineage differentiation. Conversely, donor‐derived cells are generally more mature, require fewer differentiation steps, and avoid the risks associated with residual pluripotency. Clinical‐grade iPSC manufacturing requires rigorous master‐cell‐bank characterization, assessment of genomic integrity and clonal stability, controlled differentiation, purification, and validated cryopreservation. Product‐release testing should evaluate identity, purity, lineage maturation, CAR expression, potency, genomic stability, post‐thaw recovery, and the absence or validated depletion of residual pluripotent cells. Whether these manufacturing advantages ultimately reduce total cost, improve product consistency, or translate into superior clinical outcomes remains uncertain.

Collectively, these emerging platforms illustrate a shift in non‐oncological CAR therapy from generalized enhancement of cytotoxicity toward disease‐adapted cellular engineering. Selection of cellular carriers and manufacturing strategies should therefore be guided by the dominant pathological mechanism, target‐cell location, required duration of activity, and acceptable balance between immune activation and tissue protection [111].

## 7 Common Challenges and Corresponding Strategies

### 7.1 Target‐Antigen Specificity and On‐Target/Off‐Tissue Toxicity

Target‐antigen selection remains the most fundamental bottleneck for CAR‐based therapies. An ideal target antigen should be highly enriched in pathogenic cells or pathological microenvironments, absent or expressed at low levels in normal tissues, and stably expressed throughout disease progression. However, almost no target in fibrotic or inflammatory diseases fully meets this idealized standard. FAP is highly expressed on activated fibroblasts, but also shows background expression in bone marrow mesenchymal stromal cells and certain normal pericytes; uPAR is an excellent marker of hepatic stellate cell activation, but is also expressed at low levels in some immune‐cell subsets; PDGFRβ is enriched in renal ECM‐producing cells, but is also present in normal glomerular mesangial cells; and TNF, as a pan‐inflammatory cytokine, is indispensable for systemic host defense against infection. This “on‐target off‐tissue” risk represents the foremost safety challenge for applying CAR therapy beyond oncology. FAP exemplifies the challenge of target safety in fibrotic diseases. Although enriched in activated fibroblasts, FAP is also expressed by selected stromal and repair‐associated cell populations, and preclinical FAP‐CAR studies have reported both severe toxicity and acceptable safety profiles. These divergent outcomes likely depend on CAR design, dose, persistence, antigen distribution and tissue‐repair status, underscoring the need for spatial and longitudinal validation in both diseased and normal human tissues. Antigen heterogeneity further limits both efficacy and target safety. Fibrotic lesions contain diverse stromal and immune populations whose target expression varies by organ, disease stage and patient. Broader or dual targeting may improve pathogenic‐cell coverage, but it can also enlarge the normal‐cell compartment exposed to CAR activity. Longitudinal and spatial antigen profiling is therefore required to determine whether a target remains sufficiently enriched throughout disease progression.

Multiple layers of strategies have been proposed to address this challenge. First, programmable CAR architectures can improve targeting precision: AND‐gate CARs require simultaneous recognition of two target antigens to trigger effector function. For example, an AND‐gate CAR‐M targeting both FAP and PDGFRα could theoretically restrict activity to double‐positive activated myofibroblast subsets while sparing normal repair‐associated cells expressing only FAP or PDGFRα. OR‐gated CARs respond to either antigen and may reduce treatment failure caused by heterogeneous antigen expression, although broader recognition may increase off‐tissue toxicity. NOT‐gate CARs deliver inhibitory signals upon recognition of normal tissue‐specific antigens, enabling a “lesion‐activated, normal tissue‐silent” mode of action [112]. The synNotch system creates a higher‐spatial‐precision cascade logic by using recognition of a first antigen to induce transcriptional expression of a second CAR. Split, universal and programmable (SUPRA) CARs further separate antigen recognition from intracellular signaling through soluble adaptor molecules, enabling dose‐dependent activation, antigen switching and potential pharmacological termination. However, these programmable systems remain predominantly preclinical outside oncology and face limitations including heterogeneous antigen co‐expression, circuit leakage, delayed transcriptional activation, adaptor immunogenicity and increased manufacturing and regulatory complexity. Second, affinity tuning uses intermediate rather than high‐affinity scFvs to ensure that CAR activation occurs only on cells with high antigen density, while remaining silent on normal cells with low antigen expression. Third, transient expression strategies replace viral vectors with mRNA, allowing CAR proteins to be expressed for only several days before disappearing spontaneously; this “hit‐and‐run” approach naturally avoids the cumulative off‐target risk associated with persistent CAR activity. Fourth, microenvironment‐sensing CARs provide an alternative design logic. TNF‐responsive switchable CARs do not eliminate TNF‐expressing cells, but instead rewrite TNF signaling into reparative outputs. This “sense but do not attack” strategy may reduce clearance‐related off‐target toxicity, although its long‐term impact on anti‐infective inflammatory responses still requires evaluation. Fifth, local delivery approaches, including microneedles, hydrogels, and interventional catheters, can confine CAR cells or LNPs to diseased sites and reduce systemic exposure through spatial isolation.

### 7.2 Functional Suppression of Effector Cells by the Pathological Microenvironment

The fibrotic and chronic inflammatory microenvironment imposes multidimensional functional suppression on infused CAR cells, representing a common challenge across all CAR platforms, although the specific suppressive mechanisms vary among different effector‐cell types.

#### 7.2.1 CAR‐T Cell Exhaustion

Under persistent antigen stimulation and immunosuppressive conditions, CAR‐T cells undergo a classical T‐cell exhaustion process. Inhibitory receptors such as PD‐1, LAG‐3, TIM‐3, and TIGIT are progressively upregulated, while exhaustion‐associated transcription factors such as TOX are induced, leading to reduced proliferative capacity, diminished cytokine secretion, particularly IL‐2 and TNF‐α, and impaired cytotoxic activity. In models of liver and pulmonary fibrosis, although CAR‐T cells can effectively eliminate activated fibroblasts during the early treatment phase, their effector function declines more rapidly after prolonged exposure to TGF‐β‐rich environments. Translational analyses from the phase I trial of CT‐0508 further showed that even endogenous T cells indirectly recruited by CAR‐M cells may face exhaustion pressure within chronic inflammatory microenvironments, and that the baseline exhaustion status of CD8^+^ T cells is negatively associated with therapeutic response [113, 114, 115].

Potential engineering strategies include CRISPR‐mediated knockout of inhibitory receptors such as PD‐1, as incorporated in the TyU19 design, as well as LAG‐3 or CTLA‐4; co‐expression of IL‐15 or IL‐7 to support memory‐like phenotypes and resistance to exhaustion; and metabolic reprogramming toward oxidative phosphorylation to maintain mitochondrial fitness. Armored CAR designs, such as those co‐expressing IL‐12 or IL‐18, may partially counteract TGF‐β‐mediated suppression by inducing IFN‐γ‐dependent “heating” of the local microenvironment toward a more pro‐inflammatory state. However, although armored CARs can enhance effector‐cell function, their use in non‐oncological diseases must be carefully calibrated to avoid re‐driving diseased tissues into an excessive inflammatory state [116, 117, 118].

#### 7.2.2 CAR‐M Phenotypic Plasticity, Maladaptive Repolarization and Efferocytosis Burden

The major threat faced by CAR‐M cells within fibrotic microenvironments is repolarization toward an M2‐like immunosuppressive phenotype. High concentrations of TGF‐β and IL‐10 in fibrotic tissues can directly activate Smad2/3 signaling, suppress NF‐κB activity, and downregulate the expression of pro‐inflammatory effector molecules. Meanwhile, chemotactic “find‐me” signals released by apoptotic debris, such as S1P and LPC, together with “eat‐me” signals such as phosphatidylserine, can be recognized and engulfed by CAR‐M cells, thereby triggering activation of the PPARγ/LXR pathway. Both mechanisms promote M2‐like programming. Repolarized CAR‐M cells may lose part of their anti‐fibrotic activity and, in certain microenvironmental contexts, may even secrete TGF‐β and PDGF, directly promoting fibroblast activation and collagen synthesis [119, 120]. However, macrophage plasticity may allow IL‐4Rα‐programmed M2‐like CAR‐M cells to repolarize toward an inflammatory M1‐like state in diseased tissues, potentially compromising therapeutic stability and increasing tissue injury. Direct evidence for this transition in CAR‐M cells remains limited and requires longitudinal evaluation.

Potential countermeasures include: (1) co‐expression of Legumain to enhance efferocytosis flux, reduce apoptotic debris accumulation, and thereby alleviate M2 pressure induced by persistent “eat‐me” signaling; (2) incorporation of the TLR4/TIR domain into the CAR intracellular signaling module to continuously activate NF‐κB and “lock” CAR‐M cells into a more M1‐like phenotype; (3) combination with CD47‐blocking antibodies to enhance phagocytic signaling and release CAR‐M cells from immunosuppression by blocking the CD47–SIRPα “don't eat me” axis; and (4) metabolic engineering strategies that enhance glycolytic flux to maintain M1‐associated metabolic features [121].

#### 7.2.3 Physical Barriers and Metabolic Constraints

Potential strategies to improve access to fibrotic lesions include engineering CAR‐M cells to enhance MMP‐associated ECM remodeling, co‐administration of recombinant MMPs, transient modulation of tissue permeability using low‐intensity focused ultrasound, and pretreatment with anti‐fibrotic agents such as TGF‐β inhibitors. However, nonspecific matrix disruption may impair vascular integrity or physiological tissue repair; these approaches therefore require spatial and temporal control and remain largely preclinical [59]. Hypoxia, glucose deprivation and lactate accumulation may further impair CAR‐cell function by restricting cellular metabolism and promoting mitochondrial dysfunction. Proposed countermeasures include PGC‐1α overexpression to enhance mitochondrial fitness and hypoxia‐adaptive or IL‐15‐armored designs to sustain effector activity. However, supporting evidence is derived mainly from oncology models, and their effectiveness in chronic fibrotic tissues remains to be established [122, 123].

Hypoxia, nutrient deprivation, TGF‐beta, adenosine and persistent antigen exposure can suppress or exhaust CAR‐T and CAR‐NK cells, whereas the same microenvironment may repolarize CAR‐M cells toward inflammatory or pro‐fibrotic states. Thus, imperfect specificity, heterogeneous antigen expression, restricted distribution and microenvironmental suppression are shared barriers, but platform‐specific limitations differ: CAR‐T cells risk persistent depletion and exhaustion, CAR‐NK cells have limited persistence, and CAR‐M cells combine useful tissue homing and matrix remodeling with limited proliferation and phenotypic plasticity. Preclinical uPAR‐directed CAR‐M cells have nevertheless reduced fibrosis in several liver models [62].

### 7.3 Balance Between Persistence and Controllability

Clinical translation in chronic fibrotic and immune‐mediated disease requires a balance between sufficient pathogenic‐cell depletion or tissue remodeling and preservation of immune homeostasis and physiological repair. Disease chronicity creates a persistence paradox: transient activity may permit relapse if upstream drivers remain, whereas durable CAR cells may continuously remove normal B‐lineage or repair‐associated stromal populations. The required activity window is therefore disease‐ and target‐dependent. In cancer CAR therapy, the preferred strategy is often to maximize persistence and memory formation, because residual tumor cells may relapse months or even years later. In non‐oncological diseases, however, the logic that “longer is better” needs to be reconsidered. Fibrosis provides a representative example. The reparative phase after myocardial infarction is a precisely timed process: during the early phase (0–7 days), necrotic cells and provisional matrix need to be cleared; during the intermediate phase (7–21 days), activated fibroblasts produce collagen to maintain the mechanical integrity of the ventricular wall; and during the late phase (>21 days), fibroblasts should gradually become quiescent while the ECM is remodeled into a mature scar. If CAR‐T cells are activated early in this process but persist into later stages, they may not only eliminate “excess” fibroblasts, but also interfere with matrix deposition required for late‐stage repair, theoretically disrupting scar maturation and tissue mechanical stability. A similar logic applies to autoimmune diseases: prolonged B‐cell aplasia may prevent autoantibody production, but it can also come at the cost of hypogammaglobulinemia and increased infection risk [124, 125].

Therefore, CAR therapy in non‐oncological diseases requires a design philosophy with substantially greater flexibility than that used in oncology. Several “persistence–controllability” modes have begun to emerge. First, transient modes, such as mRNA CARs lasting only several days, are suitable for acute fibrotic clearance because they are naturally reversible and carry limited long‐term safety concerns. Second, intermediate‐persistence modes, such as iPSC‐derived CAR‐NK cells lasting for weeks, may be appropriate for settings that require a certain depth of pathological cell clearance but should not persist long enough to interfere with tissue repair. Third, persistent but controllable modes, such as viral‐vector CARs equipped with safety switches including iCasp9 or tEGFR, may be suitable for systemic autoimmune diseases requiring long‐term immune resetting, provided that an “emergency brake” remains available. Fourth, persistent but naturally self‐limiting modes, as seen with autologous CD19 CAR‐T cells in autoimmune diseases, may represent a more elegant solution: after B‐cell depletion, the target antigen disappears, CAR‐T cells lose continuous activation signals, and effector cells gradually decline or enter a resting state. However, the reproducibility of this model still needs to be validated in larger‐scale trials.

### 7.4 Manufacturing Models and Accessibility Bottlenecks

The manufacturing of CAR‐cell therapies faces a fundamental “trilemma:” efficiency, low cost, and speed are difficult to achieve simultaneously. Efficiency requires sufficient cell numbers and preserved cellular function; low cost is necessary for affordability within healthcare reimbursement systems; and speed determines whether the interval from diagnosis to treatment remains clinically acceptable. Autologous CAR‐T manufacturing typically requires 2–6 weeks, and the single‐dose cost of commercially available CAR‐T products in the United States is approximately US$370 000–500 000. For patients with end‐stage fibrosis, this waiting period may translate into further irreversible loss of organ function [97].

Multiple technological routes are being explored to overcome this bottleneck. Allogeneic off‐the‐shelf strategies, represented by iPSC‐derived CAR‐NK cells and CRISPR‐edited allogeneic CAR‐T cells, aim to reduce marginal costs through the economies of scale enabled by a “one master cell bank for many patients” model. In vivo generation strategies, such as LNP‐mRNA platforms, bypass all ex vivo manufacturing steps by directly injecting nanomedicines that “assemble” CAR‐expressing immune cells inside the patient. In theory, this approach offers relatively low marginal cost and the shortest treatment initiation time; however, several challenges remain unresolved, including the scaling of delivery efficiency from rodents to large animals and humans, immunogenicity associated with repeated dosing, and precise control of the mRNA expression window. Modular universal receptor platforms, such as SUPRA‐CAR and zipper CAR systems, decouple target recognition from the effector‐cell product by using a universal CAR effector‐cell bank together with interchangeable soluble targeting adaptors, allowing target switching without the need to remanufacture the entire CAR‐cell product [29, 126].

### 7.5 Clinical Endpoint Design for Non‐Oncological Diseases

The success of CAR therapy in oncology is partly attributable to mature efficacy‐evaluation frameworks, including RECIST criteria, objective response rate (ORR), progression‐free survival (PFS), and overall survival (OS). In contrast, fibrotic and inflammatory diseases lack unified quantitative endpoints, which represents an important obstacle to clinical trial design and regulatory approval [127].

Non‐oncological CAR therapies require a multidimensional endpoint system that extends beyond oncology‐centered response metrics. Disease activity scores, such as SLEDAI‐2K, mRSS, DAS28‐CRP, and EDSS, should be combined with organ‐function indicators, including ejection fraction, FVC/DLCO, eGFR/proteinuria, and Child–Pugh or MELD scores, to capture both systemic disease control and organ‐specific benefit. Quantitative imaging, such as cardiac MRI T1 mapping and extracellular volume fraction, HRCT‐based fibrosis assessment, and liver elastography, may further evaluate structural remodeling. Molecular endpoints, including autoantibody titers, immune reconstitution profiles, and peripheral proteomic signatures, can help define the depth and durability of immune resetting. Patient‐reported outcomes, such as SF‐36 and HAQ‐DI, and hard clinical endpoints, including hospitalization, transplant‐free survival, and mortality, remain essential for long‐term evaluation. Given the delayed kinetics of immune reconstitution and tissue remodeling, follow‐up should extend for at least 1–2 years, with longer observation required for chronic fibrotic diseases.

Acute injury models in young animals incompletely reproduce the chronicity, ageing and comorbidities of human disease. Translation therefore requires chronic organ‐specific models, validation in human tissues and functional rather than solely histological endpoints. Because target‐cell depletion or reduced collagen deposition does not necessarily restore established tissue architecture or organ function, early clinical studies should prioritize patients with active, target‐positive and potentially reversible disease.

### 7.6 Special Considerations in Safety Management

Safety management of CAR therapy in non‐oncological patients differs from that in cancer patients in three key aspects. First, patients generally have more intact baseline immune function, which may allow more robust in vivo expansion of CAR cells, but may also confer better tolerance to infection, cytokine release syndrome (CRS), and immune effector cell‐associated neurotoxicity syndrome (ICANS). Second, patients usually have longer life expectancy; therefore, long‐term monitoring for secondary malignancies, with at least 5 years of follow‐up, and the impact of B‐cell aplasia on vaccine responses need to be considered from an early stage, particularly in younger patients. Third, the threshold for benefit–risk assessment is different. Patients with cancer may accept a treatment‐related mortality risk of 1%–4% in exchange for a chance of cure, whereas non‐oncological patients, even those with refractory autoimmune disease or end‐stage fibrosis, generally require a higher safety margin [128, 129, 130].

Management of immune effector cell‐associated hematotoxicity (ICAHT) may follow the risk‐stratified framework proposed by Rejeski et al. The CAR‐HEMATOTOX score is based on pretreatment hematopoietic reserve and inflammatory status, incorporating parameters such as hemoglobin, neutrophil count, platelet count, C‐reactive protein, and ferritin. High‐risk patients require close monitoring of complete blood counts, and granulocyte colony‐stimulating factor (G‐CSF) may be used in an individualized manner after CRS has stabilized or resolved, depending on infection risk and neutrophil recovery [131, 132]. Early ICAHT, occurring within 0–30 days, is mainly managed with supportive care. Late ICAHT, occurring after 30 days, should be differentiated into aplastic and intermittent subtypes: the former is associated with a more severe prognosis, higher infection‐related mortality, and may require hematopoietic stem cell boost, whereas the latter generally has a more favorable prognosis and may be managed with observation. Infection‐prevention strategies may include antiviral prophylaxis, such as acyclovir, Pneumocystis jirovecii pneumonia prophylaxis, such as trimethoprim–sulfamethoxazole, and immunoglobulin replacement during B‐cell aplasia when clinically indicated. In guideline‐consistent practice, intravenous immunoglobulin is generally considered when IgG levels fall below 400 mg/dL or in patients with recurrent or severe infections, rather than being applied indiscriminately to all patients with B‐cell depletion [130, 133] (Figure 8).

**FIGURE 8:**
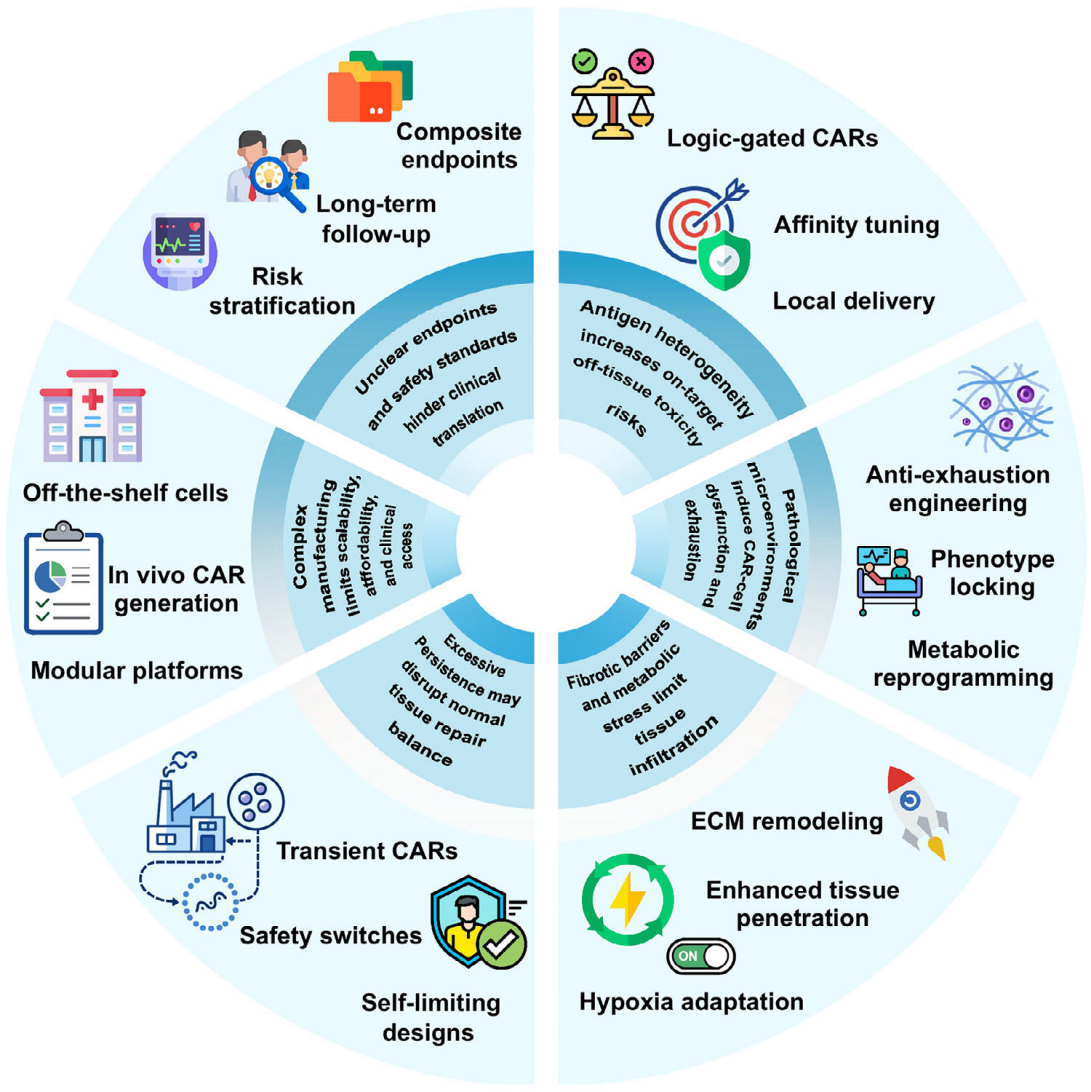
Challenges and engineering strategies for CAR‐based therapies in fibrotic and inflammatory diseases.

## 8 Conclusion and Future Perspectives

CAR‐engineered cell therapy is expanding from cancer immunotherapy toward autoimmune diseases, fibrosis, and chronic inflammation. Unlike oncology, where durable and potent cytotoxicity is often desirable, non‐oncological diseases require immune homeostasis restoration, pathological microenvironment remodeling, interruption of inflammatory–fibrotic circuits, and long‐term safety control. Therefore, CAR‐T, CAR‐M, and CAR‐NK cells should not be viewed as interchangeable or competing platforms, but as a functionally complementary matrix. CAR‐T cells are better suited for systemic immune clearance and durable immune resetting; CAR‐M cells are more appropriate for local tissue infiltration, phagocytic clearance, ECM remodeling, and inflammatory regulation; whereas CAR‐NK cells emphasize safety, controllable persistence, allogeneic manufacturing, and repeat dosing. The future of CAR therapy beyond cancer should therefore shift from simply enhancing cytotoxicity toward selecting the appropriate effector‐cell platform for a specific disease stage, tissue niche, and pathological target [85].

Next‐generation CAR development will likely converge on three major directions: intelligent design, advanced delivery, and disease‐specific strategy. At the design level, CAR systems are moving toward programmable control, context‐dependent activation, and reversible effector function through logic gating, affinity tuning, microenvironment‐responsive switches, and modular universal receptor platforms such as SUPRA‐CAR or zipper CARs. At the delivery and manufacturing level, in vivo LNP‐mRNA programming, local delivery systems, and iPSC‐derived off‐the‐shelf products may help overcome the limitations of individualized ex vivo cell production, although cell‐type selectivity, organ tropism, expression‐window control, genome stability, differentiation consistency, and release standards remain key barriers. For fibrosis and chronic inflammation, these technologies must be integrated with disease‐specific target discovery. Single‐cell, spatial, and multi‐omics approaches should be used not only to identify pan‐fibrotic markers, but also to define pathogenic cell states shaped by organ context, disease stage, and fibrotic subpopulation. Accordingly, anti‐fibrotic CAR therapy should be developed as a staged intervention system rather than a one‐time clearance strategy, combining transient CAR activity, local microenvironmental remodeling, immune resetting, and, when necessary, drugs, biomaterials, or regenerative strategies [112, 134].

Over the next 5–10 years, clinical translation is likely to proceed in a stratified manner. In the near term, randomized and prospective studies in systemic autoimmune diseases such as SLE, SSc, and inflammatory myopathies will determine whether CAR‐T therapy can move from last‐line rescue treatment to earlier intervention. In the medium term, in vivo CAR‐T, in vivo CAR‐M, and locally delivered CAR‐M platforms may enable first‐in‐human studies in selected fibrotic diseases with defined antigens, accessible lesions, unmet therapeutic needs, and quantifiable endpoints. In the longer term, iPSC‐derived CAR‐NK/CAR‐M products and modular receptor systems may improve scalability, consistency, and accessibility. Nevertheless, the field remains in transition from proof‐of‐concept to early clinical translation. Autoimmune CAR‐T evidence is still dominated by small case series, whereas fibrosis‐directed CAR‐M, CAR‐NK, and in vivo‐generated CAR‐T approaches remain largely preclinical. Future progress will depend on coordinated advances in target discovery, CAR engineering, delivery optimization, scalable manufacturing, disease‐specific clinical endpoints, regulatory approval, and reimbursement pathways. Ultimately, CAR therapy for fibrosis and inflammatory diseases should be understood not as a replacement for existing drugs, but as an immune‐engineering platform that can be integrated with pharmacological, material‐based, device‐assisted, and regenerative strategies to reset pathological immune–fibrotic programs with spatial and temporal precision [135].

## Author Contributions

**Peng Jun Xu**: Conceptualization and Writing. **Zhou Jing Zhang** and **Jing Jin**: Preparation of figures and tables. **Hai Yuan Xu**: Literature collection. **Ya Xin Gu**: Manuscript formatting. **Qian Qian Yang**: Review and Editing. **You Lang Zhou**: Supervision, Conceptualization, Review and Editing.

## Funding

This study was funded by the National Natural Science Foundation of China (82372389), Jiangsu Provincial Natural Science Foundation (BK20241841), Jiangsu Provincial Research Hospital (YJXYY202204‐ZD05) and Jiangsu provincial medical innovation center of neuromicroscopy and minimally invasive translational medicine (CXZX202212).

## Conflicts of Interest

The authors declare no conflicts of interest.

## Data Availability

The data that support the findings of this study are available from the corresponding author upon reasonable request.
